# Dining in Blue Light Impairs the Appetite of Some Leaf Epiphytes

**DOI:** 10.3389/fmicb.2021.725021

**Published:** 2021-10-18

**Authors:** Beatrix W. Alsanius, Lea Vaas, Samareh Gharaie, Maria E. Karlsson, Anna Karin Rosberg, Walter Wohanka, Sammar Khalil, Sofia Windstam

**Affiliations:** ^1^Microbial Horticulture Unit, Department of Biosystems and Technology, Swedish University of Agricultural Sciences, Lomma, Sweden; ^2^Fraunhofer IME, Computational Biology, Screening Port, Hamburg, Germany; ^3^Department of Phytomedicine, Geisenheim University, Geisenheim, Germany

**Keywords:** *Bacillus thuringiensis* serovar *israeliensis* (Bti), blue light receptor protein, light spectrum, *Pseudomonas agarici* (PA), *Pseudomonas* DR 5-09 (PDR5-09), Omnilog Phenotype MicroArray (OmniLog^®^ PM), reaction norm, *Streptomyces griseoviridis* (SG)

## Abstract

**Background:** The phyllosphere is subjected to fluctuating abiotic conditions. This study examined the phenotypic plasticity (PP) of four selected non-phototrophic phyllosphere bacteria [control strain: *Pseudomonas* sp. DR 5-09; *Pseudomonas agarici*, *Bacillus thuringiensis* serovar *israeliensis* (Bti), and *Streptomyces griseoviridis* (SG)] regarding their respiration patterns and surfactant activity as affected by light spectrum and nutrient supply.

**Methods:** The PP of the strains was examined under four light regimes [darkness (control); monochromatic light-emitting diodes (LED) at 460 nm (blue) and 660 nm (red); continuously polychromatic white LEDs], in the presence of 379 substrates and conditions.

**Results:** Light treatment affected the studied bacterial strains regarding substrate utilization (*Pseudomonas* strains > SG > Bti). Blue LEDs provoked the most pronounced impact on the phenotypic reaction norms of the *Pseudomonas* strains and Bti. The two Gram-positive strains Bti and SG, respectively, revealed inconsistent biosurfactant formation in all cases. Biosurfactant formation by both *Pseudomonas* strains was supported by most substrates incubated in darkness, and blue LED exposure altered the surface activity profoundly. Blue and white LEDs enhanced biofilm formation in PA in highly utilized C-sources. Putative blue light receptor proteins were found in both *Pseudomonas* strains, showing 91% similarity with the sequence from NCBI accession number WP_064119393.

**Conclusion:** Light quality–nutrient interactions affect biosurfactant activity and biofilm formation of some non-phototrophic phyllosphere bacteria and are, thus, crucial for dynamics of the phyllosphere microbiome.

## Introduction

The phyllosphere, i.e., plant leaves and their microenvironment, is a microbial habitat primarily colonized by bacteria and fungi (Leveau, 2006). The phyllosphere is often described as harsh, due to fluctuating water, nutrient availability, and temperature, and exposure to sunlight, in particular UV irradiation. The spatio-temporal microbial colonization pattern of leaves is highly dynamic due to non-uniform resource distribution (Beattie and Lindow, 1999; Meyer and Leveau, 2012), but also differs between locations within the plant due to leaf age and position (Ercolani, 1991; Sylla et al., 2013a,b; Alsanius et al., 2017) and over time (Sylla et al., 2013a,b). Microorganisms inhabiting the phyllosphere have developed different strategies to cope with the oscillating conditions. Among these, phenotypic plasticity (PP) and phenotypic flexibility may be strategies to succeed as an epiphytic leaf colonizer.

Nutrient availability is a key factor for an active lifestyle in the phyllosphere. Nutrients may be (i) leaked across the leaf cuticle (Leveau, 2006), (ii) lost from abiotic or biotic wounds or (iii) imported through deposition of pollen, aerial deposits, or organic debris. Relative abundances of bacterial taxa appear to be a function of plant species (Whipps et al., 2008; Knief et al., 2010), with plant leaf traits (cuticle wax composition, thickness of the cuticle wax layer, leaf age, developmental stage, and plant nutritional status) being important (Ercolani, 1991; Beattie and Lindow, 1998; Beattie and Lindow, 1999; Andrews and Harris, 2000; Leveau and Lindow, 2001; Marcell and Beattie, 2002; Lindow and Brandl, 2003; Lindow and Leveau, 2003; Leveau, 2006; Alsanius et al., 2017). These traits may also be influenced by environmental conditions, e.g., increased light intensity, decreased relative humidity, and decreased temperature (Baker, 1974).

The impact of light on the epiphytic phyllosphere bacteria has been comprehensively reviewed by Alsanius et al. (2019) but apart from responses to UV-irradiation, broad evidence and mechanisms involved in interactions between plants and crop stands, their epiphytic microbiome and light wave length are lacking. However, from a plant perspective, short-wave blue (425–475 nm) and long-wave red (625–675 nm) parts of the visible light spectrum are important for photosynthesis, an essential motor of nutrient availability on the leaf surface. Certain light bands may also affect the microbial community structure (Alsanius et al., 2017), including survival and lifestyle of some non-photosensitive microorganisms, as displayed in recent literature (Gomelsky and Hoff, 2011; Ondrusch and Kreft, 2011; Wu et al., 2013; Alsanius et al., 2019). In a recent *in vitro* study, we demonstrated light spectrum-dependent utilization of nutrient sources by the phyllosphere-colonizing bacterium *Pseudomonas* sp. DR 5-09 (Gharaie et al., 2017a). The finding that respiration of 372 substrates and conditions were distinctly affected by monochromatic blue LEDs as opposed to monochromatic red LEDs and exposure to darkness during incubation sheds light on the potential complexity of phyllospheric microbial lifestyle decisions.

Phenotypic plasticity is defined as “environmentally sensitive production of alternative phenotypes by given genotypes” (DeWitt and Scheiner, 2004). The PP model described by DeWitt and Scheiner (2004), which discriminates between responses (*reaction norms*) based on genetic (G) and environmental (E) factors and their interactions (GxE), was used in the present analysis. To study the impact of light spectrum on PP, we employed a method for leaf-independent responses recently developed in a study which exposed the model organism *Pseudomonas* sp. DR 5-09 (PDR5-09) to four light regimes [dark incubation (default) and incubation under blue, red and white LEDs] (Gharaie et al., 2017a). To determine whether the outstanding effect of blue light might be a general effect on phyllosphere bacteria and to explore the importance of PP in coping with phyllosphere conditions, we added an additional dimension, i.e., impact, to the two-dimensional DeWitt and Scheiner model (DeWitt and Scheiner, 2004) in this study. We used three bacterial strains *Pseudomonas agarici* (PA), PDR 5-09, *Bacillus thuringiensis* serovar *israelensis* (Bti) isolated from the phyllosphere of greenhouse-grown ornamental species, all of which exhibit *in vitro* inhibition of mycelial growth of *Botrytis cinerea*, *in vitro* protease and chitinase activity and biosurfactant formation, plus a commercial biocontrol strain, *Streptomyces griseoviridis* (SG), displaying the same properties in an *in vitro* screening. These four strains are named target strains. Dark incubation was considered a baseline (environment A) and three light spectra were used as alternative environments (environment B). The aggregated substrate utilization properties of the four strains denoted reaction norms to different light environments. Environmental stresses originating from restricted nutrient availability and exposure to certain monochromatic light bands among phyllosphere bacteria are already a reality for the modern high-technology horticultural industry, which is currently undergoing a major shift in lighting technology (Nelson and Bugbee, 2014). In this context, PP and flexibility are critical mechanisms for the indigenous phyllosphere microbiota and microorganisms actively or passively immigrating to leaf habitats, such as leaf pathogens and biological control agents (Wilson and Lindow, 1993). Successful leaf colonizers must be able to adapt to the prevailing environmental conditions, but also to actively prospect for nutrient-rich sites. In this regard, an ability to form surface-active compounds (biosurfactants), reducing the tension on surfaces and interfaces occurring on the waxy leaf cuticle during the stationary phase, is an advantage. A common feature of biosurfactants is their amphiphilic character, i.e., consisting of one hydrophobic and one hydrophilic end, but from a chemical perspective they are a heterogeneous group, e.g., glycolipids, lipopeptides, phospholipids, fatty acids, neutral lipids, polymeric, and particulate compounds (Mulligan, 2005). Biosurfactant formation has been reported for *Pseudomonas*, *Bacillus*, and *Streptomyces* (Guerra-Santos et al., 1986; Morikawa et al., 1992; Hue et al., 2001; Kuiper et al., 2004; Raaijmakers et al., 2006; Rivardo et al., 2009; Hultberg et al., 2010a; Trevisan Slivinski et al., 2012; Kalyani et al., 2014; Manivasagan et al., 2014), and the four strains used in this study all displayed this property in *in vitro* screening.

Our starting hypotheses were that: (i) light spectrum affects the PP of epiphytic phyllosphere colonizers; (ii) blue, but not red, light impairs the respiration pattern of target strains; and (iii) the response of bacterial strains to nutrition and light conditions is reflected in their ability to form biosurfactants.

## Materials and Methods

### Microbial Procedures

*Pseudomonas agarici* Ib1FK (strain NCPPB 2472; query cover: 100%; *E*-value: 2e^–64^; Identification: 100%), *Pseudomonas* sp. DR 5-09 (PDR5-09) Ib5FK (Gharaie et al., 2017a) and *Bacillus thuringiensis* serovar *israelensis* (Bti) PCb52T (strain AM65-52; query cover: 100%; *E*-value: 0; Identification: 100%) were all isolated from greenhouse-grown ornamentals and in a previous screening showed protease and chitinase activity and biosurfactant formation. They also inhibit mycelial growth of *Botrytis cinerea in vitro* (Gharaie et al., 2017b). *Streptomyces griseoviridis* (SG; CBS 904.68) was purchased from Centraalbuureau voor Schimmelcultures, Utrecht, Netherlands. All strains were propagated in full-strength tryptic soy agar (TSA; DF 0369-17-6; Difco Laboratories Inc., Detroit, United States) and incubated overnight at 30°C, before being prepared for phenotypic microarray measurements.

The phenotypic microarrays (PM) were performed on two carbon source panels (PM01 and PM02), and on nitrogen (PM03), and phosphorus, and sulfur (PM04) substrate panels (Biolog Inc., United States, catalog nos. 12111, 12112, 12121, and 12131, respectively), providing 190 carbon (C) sources, 95 nitrogen (N) sources, 59 phosphorus (P) sources, and 35 sulfur (S) sources. The composition of substrates on the panels is shown in Supplementary Table 1. Substrate concentrations differ among the four panels as regard carbon sources (2–20 mM), nitrogen sources (1–5 mM), phosphorus sources (0.1–1 mM) and sulfur sources (0.1–1 mM) (B. Bochner, personal information). The C sources (PM01 and PM02) are incubated as sole substrates, whereas the N, P, and S source panels (PM03 and PM04) are supplemented by 2 mM sodium succinate and 2 μM ferric citrate as additional carbon sources (enrichment). In addition to C utilization, 37 compounds (referred to as “co-catabolic substrate subset”) serve as model substrates to study their impact as N sources (L-alanine, L-arginine, L-asparagine, L-aspartic acid, L-cysteine, L-glutamic acid, L-glutamine, glycine, L-histidine, L-isoleucine, L-leucine, L-lysine, L-methionine, L-phenylalanine, L-proline, L-serine, L-threonine,L-valine, D-alanine, D-aspartic acid, D-serine, L-homoserine, L-ornithine, *N*-acetyl-L-glutamic acid, L-pyroglutamic acid, putrescine, tyramine, acetamide, glucuronamide, D-glucosamine, *N*-acetyl-D-glucosamine, *N*-acetyl-D-galactosamine, adenosine, thymidine, uridine, and inosine), P sources (D-glucose-1-phosphate, D-glucose-6-phosphate), and S sources (L-cysteine, L-methionine) sources. In the present study, the PM assays were performed according to the standard protocols of the manufacturer for Gram-negative bacteria (*Pseudomonas*) and Gram-positive bacteria, as previously described in detail (Gharaie et al., 2017a). In brief, colony swabs were used to harvest cells of the four strains from overnight cultures, which were then suspended in IF-0a GN medium (Biolog Inc., Hayward, CA, United States). The turbidity of the bacterial suspension was adjusted turbidimetrically (Biolog Inc., United States, catalog no. 3587) to 85 and 81% transmittance for Gram-negative and Gram-positive strains, respectively, before addition of the redox dye (Gram-negative strains: Dye mix A, catalog no. 74221; Gram-positive strains: Dye mix F, catalog no. 74226; Biolog Inc., Haywood, United States). A 100 μl aliquot of the suspension was pipetted into each plate well. Thereafter, plates were sealed with Greiner ViewSeal (Greiner Bio-one, 676070; Sigma Aldrich, Z617571-100EA, St. Louis, MO, United States) for 96-well plates. Based on light spectra of interest for horticultural plant production, the panels were exposed to three light regimes, namely monochromatic LEDs (blue: 460 nm; red: 660 nm) and polychromatic white LEDs covering 350–990 nm) as previously displayed in Gharaie et al. (2017a). Control panels were incubated in darkness. Panels exposed to darkness were kept at 20°C in the OmniLog reader (OmniLog, catalog no. 93182, Biolog Inc., United States) during the entire incubation period, with 15 min measurement intervals over a 96 h period. Light exposure took place in lined cabinets (500 mm × 500 mm × 1,000 mm), which were arranged in a climate room (constant temperature: 20°C) with eight plates running simultaneously. Each cabinet was equipped with LED lights (90 W, Trädgårdsteknik AB, Ängelholm, Sweden), the spectral output of which was measured using a spectroradiometer (Li-Cor Li-1800, Li-Cor, Lincoln, NE, United States), and light intensity was adjusted to 100 μmol m^–2^ s^–1^ by adjusting the distance between the light source and the PM plates. Utilization of the different substrates was monitored as color changes in the redox dye tetrazolium blue from colorless to purple. Color change of LED-exposed panels was measured by repeated short readings using the Omnilog reader at distinct time points recording for 1 h. The reading times for the two *Pseudomonas* strains and Bti were at 0, 7, 14, 21, 24, 28, 36, 42, 48, 54, 60, 66, 72, 78, 84, 90, and 96 h of incubation and for S, at 0, 7, 14, 21, 24, 28, 36, 42, 48, 54, 60, 66, 72, 78, 84, 90, 96,108, 120, 132, 144, 156, 168, 180, and 192 h of inoculation.

### Biosurfactant Formation

Biosurfactant formation was assessed using the drop collapse test previously described by Youssef et al. (2004). For this purpose, microtiter plate templates (1.4 cm × 1.4 cm) were fixed between glass plates lined with parafilm. Aliquots of 20 μl were transferred with an eight-channel pipetter (E1-ClipTip Equalizer 8×, 2–125 μl, Thermo Fisher Scientific #4672050) and, after adjustment of the pipette tip distance, dripped on the lined glass plate. Droplets were ranked from 0 to 2 (0: convex droplet; 1: moderately convex droplet; 2: flattened droplet) (Supplementary Figure 2).

### Biofilm Quantification

For endpoint biofilm quantification, the PM 1 and 2 wells from plates incubated with PDR5-08 and PA were emptied from all remaining liquid. Remaining unattached cells and tetrazolium dye residues were removed in a two-step-washing approach, consisting of (i) repeated imbibing each well of the Omnilog panels with 100 μl of water for 5 min (two cycles) and (ii) gently submerging the individual panels in a small tub of water as a last cleaning step followed by removal of all liquid. Biofilm staining was facilitated by adding 100 μl of 0.1% solution of crystal violet to each well. After 20 min of incubation, the dye was removed and washed as described above, using three cycles of the washing step. The plates were then left to dry in the fume hood for 30 min. Finally, 100 μl of 96% ethanol was added to each well and left for 1 h before the plates were read spectrophotometrically (Expert 96^TM^ spectrophotometer, AsysHiTech, Eugendorf, Austria).

### Molecular Identification of Blue Light Receptors

The three isolated strains, *Pseudomonas agarici* (PA), *Pseudomonas* DR5-9 (PDR5-09), and *Bacillus thuringiensis* serovar *israeliensis* (Bti) were sent to LGC, Berlin, Germany for whole genome sequencing with Sanger/Illumina 1.9. Computational work was performed on resources provided by SNIC through Uppsala Multidisciplinary Center for Advanced Computational Science (UPPMAX) in order to extract amino acid sequences of the superfamily PAS domain. Each sequence was blasted in a BLASTP search^1^ for identification of the blue light photo receptor gene. The sequences were aligned and analyzed in BioEdit 7.2.5 (Hall, 1999).

### Calculation and Bioinformatics

After data recording and export to .csv-files using OmniLog^®^ PM kinetic analysis software (Product Number UA24331-PMM, version 1.6), all further data management steps, graphical representations, and statistical analyses of PM data were performed using R (R Core Team, 2016) and functionality from the dedicated R package opm (Vaas et al., 2012a) as previously described by Gharaie et al. (2017a). Manual curation led to exclusion of the following substrates for statistical analysis: on plate PM01 D-maltose, maltotriose, L-rhamnose, D-cellobiose, sucrose, D-glucose-6-phosphate, D-trehalose, D-psicose, L-fucose, D-fructose-6-phosphate, adonitol, and a-D-glucose-1-phosphate; and on plate PM02 b-gentiobiose, palatinose, xylitol, turanose, D-arabinose, L-arabitol, and b-methyl-D-uylopyranoside. Raw data were arranged and maximum curve height was calculated as previously described by Vaas et al. (2012b). For visualization, functionalities from dedicated package opm (Vaas et al., 2012b) for heatmaps and package Vennerable (Swinton, 2011) for Venn diagrams were used. Total utilization (total number of omnilog units *n*), dominance ($\sum_{\text{i}}{\left(\frac{n_{\text{i}}}{n}\right)}^{2}$, with *n* = number of total omnilog units and *n*_i_ = number of omnilog units in substrate *i*), ratio utilization of the dominant compound and total number of omnilog units were calculated using PAST vers. 3.14.^2^ Impaired substrate utilization patterns were compared with biochemical pathways using the Kyoto Encyclopedia of Genes and Genomes (KEGG)^3^ to identify the probable metabolic impact of light spectra. KEGG-pathway maps were drawn using functionality provided by package pathview (Luo and Brouwer, 2013). Biosurfactant formation was analyzed using principal component analysis (PCA) on the basis of all recorded data for two replicate samples employing PCA methods in R and dedicated visualization provided by package ggbiplot (Vu, 2011).

## Results

The results are presented on overall level and for small-scale analysis of subsets. Comparisons with the responses by the strains to light and reaction norms are panoramically portrayed and substrate utilization patterns of potentially affected metabolic pathways are mapped. Sole and co-catabolic (enriched) substrate utilization are compared and correlated to biosurfactant formation.

### Comparison of Bacterial Substrate Utilization Patterns

**FIGURE 1:**
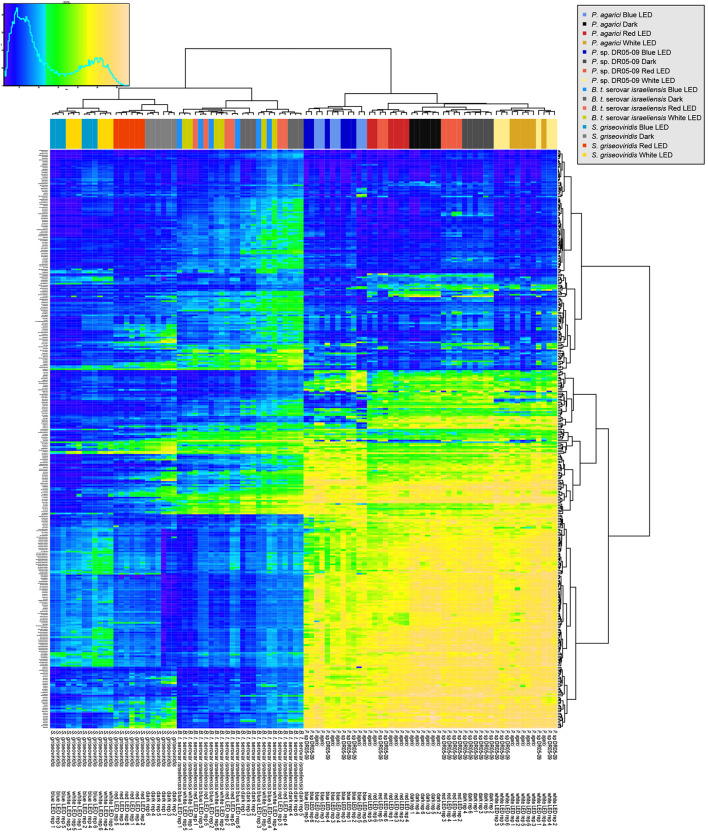
Overview of respiration behavior of *Pseudomonas agarici* (strain 1), *Pseudomonas* sp. DR 5-09 (strain 2), *Bacillus thuringiensi*s serovar *israeliensis* (strain 3), and *Streptomyces griseoviridis* (strain 4) on 190 carbon (C), 95 nitrogen (N), 59 phosphorus (P), and 35 sulfur (S) sources. The heatmap displays maximum curve height values monitored during 96 h (strain 1–3) and 192 h (strain 4) when exposed to blue, red, or white LEDs or darkness. The legend (*upper left corner*) explains the color code from blue to green, while yellow shades indicate low, moderate, and high substrate utilization, assessed as arbitrary Omnilog values. The histogram describes the frequency of maximum height reached for the various substrates.

When incubated in darkness (the default mode for Phenotype MicroArrays^®^), decisive utilization patterns were found for the Gram-negative and Gram-positive organisms tested, with the most distinct separation between strains when exposed to C sources. With increasing substrate richness (N, P, and S sources), Gram-negative and Gram-positive clusters were maintained, but the test organisms within these clusters displayed less strain-specific utilization patterns. Substrate preferences of the two *Pseudomonas* strains became more similar under monochromatic or polychromatic LEDs, irrespective of substrate richness (Figure 1).

### Blue Light Receptors

**FIGURE 2:**
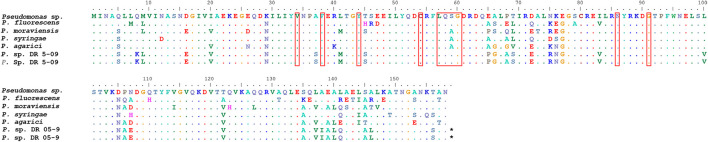
Alignment of putative blue light receptor gene of *Pseudomonas* spp. in comparison with sequence from NCBI accession number WP_064119393. Putative active sites are marked with squares.

Three sequences of the putative blue light receptor gene were identified, one in PA and two in PDR5-09, which were all identical and showed 91% similarity with the sequence from NCBI accession number WP_064119393 (Figure 2). Nine conserved active site residues of the blue light receptor were also found (34 V, 38 F, 44 Y, 57 L, 58 Q, 59 S, 60 G, 86 N, and 91 G) (Figure 2). The BLAST results confirmed that these sequences belong to the PAS superfamily, which has been found to act as sensor for light and oxygen in signal transduction (Ponting and Aravind, 1997). For SG, neither putative blue light receptor genes nor PAS domains could be identified. In total, nine amino acid sequences were found in Bti identified as PAS domain-containing protein. All sequences had the PAS domain followed by either histidine kinase or GGDEF domain. However, only one sequence had the PAS domain followed by the STAS superfamily, which regulates stress and is characteristic for blue light receptor in *Bacillus* sp. (NCBI accession number WP 004399022). The putative active sites revealed the same structure as the blue light receptor in *Pseudomonas* sp. with nine active sites. However, both amino acids and the positions seem not to be conserved as it varied between sequences.

### Light-Dependent Reaction Norms

**FIGURE 3:**
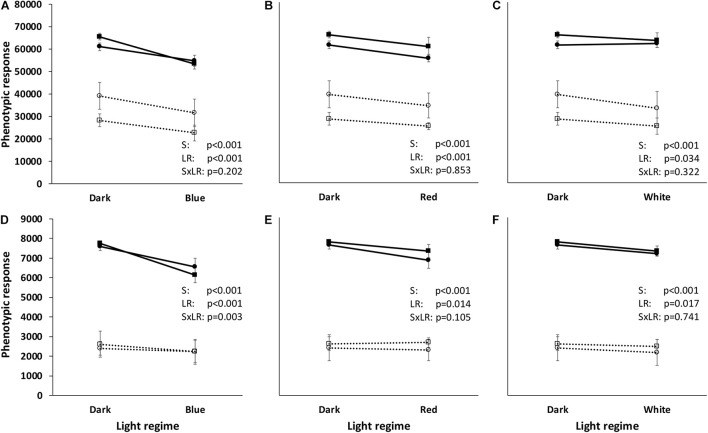
Reaction norms of two Gram-negative (*Pseudomonas agarici*: black line, black-filled circles; *Pseudomonas* sp. DR 5-09: black line, black-filled squares) and two Gram-positive (*Bacillus thuringiensis* serovar *israeliensis*: black broken line, black-open circles; *Streptomyces griseoviridis*: black broken line, black-open squares) bacteria when exposed to different light regimes. The reaction norms are based on the phenotypic response to all 384 test substrates **(A–C)** and 37 test substrates (“co-catabolic subset”) **(D–F)** extracted from phenotypic microarrays using PM01-PM04 (Biolog, Haywood CA, United States). Total utilization was assessed by kinetic measurements of color change in the redox dye tetrazolium blue and calculated on the basis of maximum curve height (Omnilog units). Each value point corresponds to six independent replicates. Statistical information is based on ANOVA (general linear model) followed by Tukey’s test (p < 0.05) with respect to strain (S), light regime (LR), and their interaction (SxLR).

Total substrate utilization based on the maximum curve height of each individual substrate varied between the four strains and the four light regimes. Irrespective of light regime, the highest overall utilization was found for the two Gram-negative strains (PDR5-09 > PA), whereas overall utilization of the two Gram-positive strains was approximately 57% (Bti) and 42% (SG) of that in the control strain (*p* < 0.001). Beside these differences between the organisms, we found significant overall light effects between the four light regimes (dark > red > white > blue) with respect to total utilization (*p* < 0.001). Although utilization by the strains was evenly distributed, significant differences were noted between the Gram-negative and Gram-positive strains (*p* < 0.001). Likewise, the ratio between the dominantly utilized substrate and the sum of Omnilog units differed significantly between the Gram-positive and Gram-negative strains (*p* < 0.001), leading to differences in strain × light regime interactions (*p* = 0.002). Overall analysis of the utilization pattern of these sources displayed a significant effect of exposure to blue LED, with utilization being 10–16% lower than under the dark, red, or white LED regime. The severest impact was found on the control strain under blue LEDs when all compounds were included in the analysis and both strain-dependent and light spectrum-dependent effects were found (Figures 3A–C). The reaction norms were more pronounced on scrutinizing the co-catabolic substrate subset (Figures 3C–F and Table 1) and strain × light treatment interactions were found when comparing the dark and blue LED-incubated samples. Although the two *Pseudomonas* strains were genetically different, they displayed similar plastic responses when exposed to the selected light regimes with respect to substrate utilization and putative biosurfactant formation. Interestingly, increasing substrate richness reduced the differences known from control dark conditions between ecotypes.

**TABLE 1:** Substrate utilization of selected sources (“co-catabolic substrate subset”) by two Gram-negative (*Pseudomonas agarici*; *Pseudomonas* sp. DR 5-09) and two Gram-positive (*Bacillus thuringiensis* serovar *israeliensis*; *Streptomyces griseoviridis*) bacterial strains exposed to four light regimes during incubation (dark incubation, incubation under short-wave monochromatic blue, long-wave monochromatic red and continuous polychromatic white light-emitting diodes (LEDs) at 20°C.

		Dark	Blue LEDs	Red LEDs	White LEDs
*Pseudomonas agarici*		7,599.67 A^1^	6,557.33 BC	6,839.83 ABC	7,174.00 AB
*Pseudomonas* sp. DR 5-09		7,763.00 A	6,128.00 C	7,287.17 AB	7,297.83 AB
*Bacillus thuringiensis* serovar *israeliensis*		2,404.33 D	2,243.17 D	2,296.50 D	2,176.50 D
*Streptomyces griseoviridis*		2,613.50 D	2,230.67 D	2,673.83 D	2,488.33 D

**Source**	**DF**	**Adj SS**	**Adj MS**	***F*-Value**	***p*-Value**

Strain	3	5.29E+08	1.76E+08	795.81	<0.001
Light regime	3	7,964,689	2,654,896	11.99	<0.001
Strain × Light regime	9	5,264,305	584,923	2.64	0.010
Error	80	17,711,547	221,394		
Total	95	5.6E+08			

*The experiment was conducted using phenotypic microarray panels PM01-04 (Biolog Inc., Haywood, CA, United States) with tetrazolium chloride as an indicator for respiration. Substrate utilization was registered in a computer-integrated camera system (Omnilog), expressed in Omnilog units and calculated on the basis of maximum curve height. Data analysis based on general linear model included light regime and strains as factors as well as their interactions and was followed by Tukey test (*p* < 0.05). ^1^Mean values followed by the same letter do not differ significantly based on GLM followed by Tukey test (*p* < 0.05).*

### Substrate Utilization

Substrate utilization by strain PDR5-09 has previously been described in detail (Gharaie et al., 2017a) and it served here as the control strain. Together with the three other strains tested (PA, Bti, and SG) four distinct response patterns to light regime with respect to substrate utilization were displayed (Figure 1):

1. Blue and white LEDs differed from red LEDs and dark incubation (PA: C sources, SG: C sources, N sources, P, and S sources).
2. Blue and red LEDs differed from white LEDs and dark incubation (PA: P and S sources).
3. Dark incubation differed from all LED regimes (PA: N sources).
4. No response to light regime (Bti: all sources tested).

In addition, strain-specific differences were found for the different substrate × light interactions.

**FIGURE 4:**
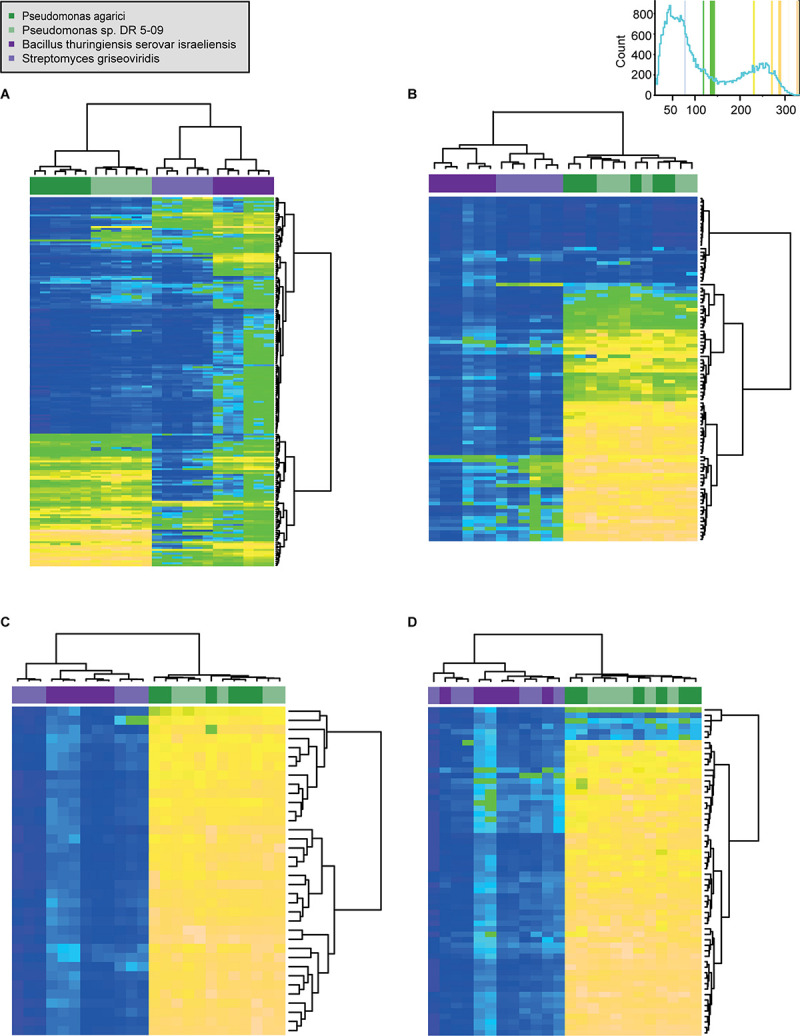
Comparison of respiration behavior of *Pseudomonas agarici* (dark green), *Pseudomonas* sp. DR 5-09 (light green), *Bacillus thuringiensis* serovar *israeliensis* (purple), and *Streptomyces griseoviridis* (light purple) on 190 C **(A)**, 95 N **(B)** 35 S **(C)**, and 59 P **(D)** sources during dark incubation. The heatmap displays maximum curve height values [monitored during 96 h (both *Pseudomonas* and *B. thuriengiensis*) and 192 h (*S. griseoviridis*)]. The legend (*upper right corner*) explains the color code from blue to green, while yellow shades indicate low, moderate, and high substrate utilization, assessed as arbitrary Omnilog values. The histogram describes the frequency of maximum height reached for the various substrates. Detailed information about the substrate names and orders are available in Supplementary Material 3.

Differences between strains did not depend on light regime. The overall comparison (Figures 4A–D and Supplementary Material 3) showed different utilization patterns of the four groups of substrates. Blue LEDs affected the substrate respiration of the two *Pseudomonas* strains distinctly and significant differences were found for the other three light regimes. In contrast, no effect of light regime was found with respect to substrate utilization by Bti. Substrate utilization by SG clustered into two groups: (i) dark and red LED incubation and (ii) white and blue LED incubation.

Preferred and consistently used substrates differed between the four strains and light treatments. The two Gram-negative strains had cravings for L-hydroxyproline. However, the most pronounced and consistent preferences were of PA under red LEDs, for L-hydroxyproline, L-histidine, and alanine-glutamine. Putrescine was the only substrate consistently utilized under blue LEDs, but only in the case of PDR5-09. One (gelatin) and two substrates (D-ribose, L-lyxose) were consistently highly desired by Bti and SG, respectively, when exposed to all four light regimes. Consistent color change at high levels only under blue LEDs was found for D-ribose in the case of Bti and for sodium pyrophosphate and trimetaphosphate in the case of SG. However, the greatest extent and most consistent utilization (top 10 substrates) was found for the two Gram-positive strains under dark conditions. Scrutiny of the co-catabolic substrate subset showed distinctly higher utilization (by almost 300%) of these energy sources by the two Gram-negative compared with the Gram-positive strains.

### Comparison of Light Spectrum-Dependent Substrate Utilization Between the Two *Pseudomonas* Strains

Light-spectrum dependent differences between the two *Pseudomonas* species are presented in Table 2. Irrespective of light regime, β-cyclodextrin and gelatin were utilized to a significantly higher extent by the control strain than by PA. The differences were most pronounced for C sources when exposed to dark conditions and for S sources under red LEDs. Differences in substrate utilization between the two strains diminished under blue LED incubation. No significant differences in respiration pattern were found between the strains for dark-incubated N, P, and S sources, or for white and blue LED-exposed S sources.

**TABLE 2:** Comparison of substrate utilization by the control strain and *Pseudomonas agarici*, analyzed by *T*-test based on six individual replicates.

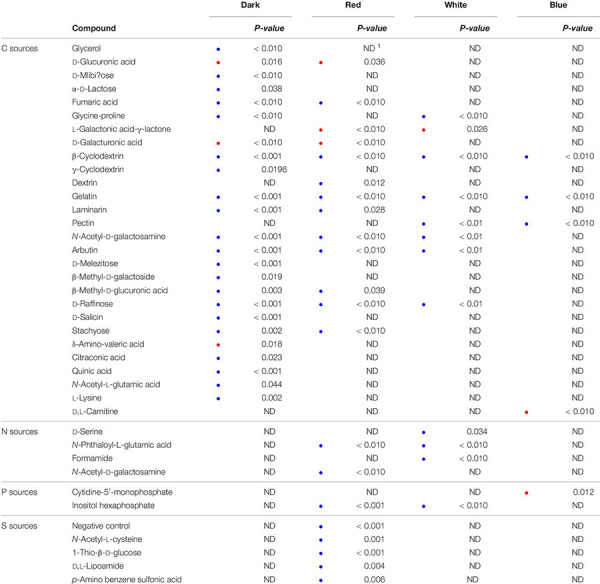

*Substrates and *p*-values are displayed. Blue dots denote significantly higher respiration by the control strain, red dots higher utilization by *P. agarici*. ^1^Absence of significant differences is marked as no difference (ND).*

### Interaction Between Substrate Enrichment and Light Regime

Enrichment and light regime had a significant impact on substrate utilization. However, six sources (L-homoserine, tyramine, thymidine, *N*-acetyl-D-galactosamine, D-aspartic acid, acetamide) resisted utilization under all conditions. A general influence of enrichment was found for five N substrates (*N*-acetyl-β-D-mannosamine, glucuronamide, L-lysine, glucose-1-phosphate, and glucose-6-phosphate) and two P substrates (uridine, *N*-acetyl-D-glucosamine), which displayed a substantial increase and slight decrease in utilization when serving as an N and P source, respectively. The importance of form of enrichment was demonstrated by L-methionine, which abolished irrespective of light regime when serving as a C or N source, but significantly utilized as an S source. The heatmap comparing utilization of the 37 substrates that served both as sole C sources and as N, P, or S sources (co-catabolic substrate subset) separated the blue LED treatment from the other three light regimes for PA. For example, utilization of L-phenylalanine and L-valine offered as a sole C source was low when exposed to blue LEDs, but efficiently utilized when enriched irrespective of light regime. In contrast, L-histidine was exclusively used by PA as a C source under blue LEDs, but efficiently respired in all light treatments when enriched. L-isoleucine was not respired when exposed to blue LEDs irrespective of enrichment, as opposed to all other light regimes. Light × enrichment interactions were found for three amino acids when offered as N sources: (i) D-serine, showing increased utilization under blue and white LEDs, (ii) L-threonine, increasingly utilized under dark conditions and red LEDs and (iii) glycine, downtuned in the presence of blue LEDs, but efficiently utilized under red and white LEDs and in darkness.

No light spectrum effects were observed for utilization of sole or enriched substrates by Bti. However, two utilization patterns were identified. Pattern A separated the substrates with respect to their general respiration, into (i) not utilized irrespective of the richness of the substrate (L-homoserine), (ii) variable utilization response or (iii) consistently utilized. Pattern B grouped the compounds with respect to their response to enrichment into: (i) increased utilization when enriched, (ii) moderate decrease when enriched, or (iii) severe decrease in utilization when enriched. Interestingly, most of the substrates were substantially better utilized when offered as sole C sources and the highest and most consistent utilization was found for L-histidine, L-threonine, L-aspartic acid, L-proline, L-glutamic acid, L-alanine, L-asparagine, thymidine, D-glucosamine, L-glutamine, D-serine, uridine, *N*-acetyl-D-glucosamine, D-glucose-6-phosphate, L-serine, adenosine, and inosine. Utilization was maintained at a variable level for a few substrates when enriched (α-D-glucose-1-phosphate, L-histidine, L-asparagine, L-threonine, L-ornithine, L-glutamine), whereas respiration of a majority of enriched substrates ceased.

The impact of light regime on substrate utilization by SG was less pronounced than for PA, but also less scattered than for Bti. Blue LEDs influenced the respiration pattern, but not distinctly differently from the white LEDs. However, respiration of the substrates serving either as sole C or enriched N, P, or S sources, in general, was limited. Five horizontal clusters were identified. In all light regimes, L-histidine was abolished when serving as a C source and increased, although inconsistently, when offered as an N, P, and S substrate. Respiration of D-aspartic acid, D-alanine, uridine, L-aspartic acid, glucuronamide, D-serine, *N*-acetyl-D-galactosamine, L-proline, *N*-acetyl-D-glucosamine, and D-glucosamine ceased when offered enriched. Respiration of α-D-glucose-1-phosphate and D-glucose-6-phosphate by SG slightly increased under blue and white LEDs. Likewise, L-aspartic acid, L-glutamine, and glycine were not utilized as sole substrate in any of the light regimes, but enrichment inconsistently increased under dark incubation and red LEDs. A particular respiration pattern was found for *N*-acetyl-D-glucosamine and D-glucosamine, which were utilized efficiently, respectively, under dark and red LEDs, abolished under blue and white LEDs when serving as sole C sources, and utilization was suppressed completely under enriched conditions. Similar, but less pronounced, behavior under co-catabolic (enriched) conditions was displayed in the presence of L-proline. L-glutamic acid was utilized by SG and efficiently under the dark and red LED regime, respectively, with slight differences in dependence on substrate richness. The importance of the configuration of substrate richness was also reflected in utilization of SG; cysteine serving as N source was respired under dark incubation and red LEDs, but completely suppressed as an S source.

### Biosurfactant Formation as a Response to Nutrient Availability and Light Spectrum

**FIGURE 5:**
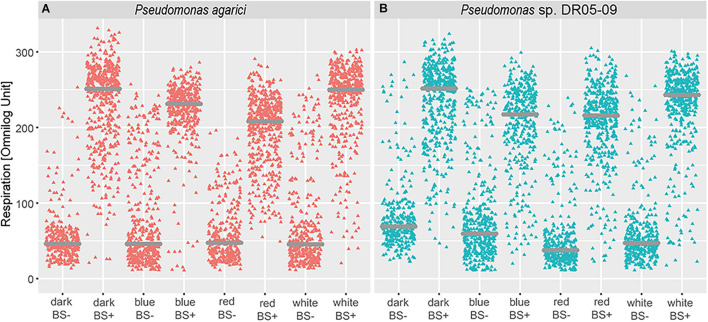
Impact of light regime (dark incubation or incubation under blue, red, or white LED light) on biosurfactant formation of *Pseudomonas agarici* and *Pseudomonas* sp. DR 5-09. **(A,B)** display mean substrate utilization (expressed as Omnilog values) depending on surface activity of the suspensions (using drop collapse test) at the end of the incubation period by *P. agarici* and *Pseudomonas* sp. DR 5-09, respectively. BS+ and BS- indicate positive and negative biosurfactant formation, respectively.

Irrespective of light regime, drop collapse was noted only for few substrates incubated with the Gram-positive strains and the results were inconsistent. In contrast, in the case of the *Pseudomonas* strains, surface activity was clearly influenced by the light regime. Thus, data analysis was limited to the two *Pseudomonas* strains. In general, a positive drop collapse test response was predominantly found for substrates that were intensively respired and mean values of respired compounds with surface activity were higher when exposed to dark incubation and white LEDs than to blue and red LEDs (Figure 5). Mean utilization rates, assessed as maximum curve height, were significantly different between strains with and without surface activity, irrespective of light regime, for both *Pseudomonas* strains. Both strains displayed similar reaction norms to different light regimes with respect to mean utilization rate, but qualitative differences occurred with respect to substrate (Figure 4 and Table 3). However, differences occurred with respect to individual substrates and the default incubation mode (dark incubation) did not predict the potential for surfactant production. Under dark conditions, both strains produced biosurfactants when grown on the following sole C sources: *N*-acetyl-D-glucosamine, succinic acid, D-galactose, glycerol, L-lactic acid, D-galactonic acid-γ-lactone, D,L-malic acid, D-ribose, acetic acid, D-glucosaminic acid, and α-keto-glutaric acid. No production was detected in the presence of L-fucose, D-glucose-6-phosphate, lactulose, and sucrose and drop collapse was absent in Tween 20 and Tween 40. The dark-incubated strains differed with respect to biosurfactant formation when exposed to D-trehalose, D-xylose, L-rhamnose, and D-maltose (PDR5-09 > PA), and D-glucuronic acid, α-D-lactose, and uridine (PA > PDR 5-09).

**TABLE 3:** Impact of light regime and substrate on putative biosurfactant formation by *Pseudomonas agarici* (normal font) and *Pseudomonas* sp. DR 5-09 (italics) as assessed by drop collapse test.

Sources	Group 1	Group 2	Group 3	Group 4	Group 5
N	Glycine-glutamic acid, *m-tartaric acid*, caproic acid, L-isoleucine, L**-valine**, L*-leucine*	Succinic acid, D,L*-malic acid*, glycerol, D-galactonic acid-g-lactone, **acetic acid**, D*-maltose*, D*-glucosaminic acid*, and α*-keto-glutaric*	D-glucoronic acid, **4-hydroxy-benzoic acid**, malonic acid, and *caproic acid*	L*-isoleucine*, *citraconic acid*	L-arabinose, α-keto-valeric acid, 2-hydroxy-benzoic acid, and γ*-hydroxy-butyric*
P	D**-asparagine**, *sodium nitrite*, *sodium nitrate*, *glycine*, L*-isoleucine*,L*-valine*, and D*-lysine*	Sodium nitrite, sodium nitrate, glycine, L-isoleucine, D-lysine, ethanolamine, cytosine, thymine, uracil, δ-amino-valeric acid, glycine-methionine, *adenine*, and *guanine*	L **-threonine**		
S	Cysteamine-*S*-phosphate, *parabanic acid*, *thymidine-3′-monophosphate*	Negative control, **trietyl phosphate**, **hypophosphite**, phosphor-glycolic acid, cytidine-5′-monophosphate, phosphor-L-arginine, **phosphono acetic acid**, **methylene diphosphonic acid**, thymidine-5′-monophosphate, *Allantoin*, D-2-phospho glyceric acid			

*The compounds in bold font had the same reaction norm for both strains (Group 1: Absence of drop collapse at lower respiration rates when exposed to blue LEDs compared with the other light regimes; Group 2: Absence of drop collapse when exposed to blue LEDs, but respiration rates comparable between the four light regimes; Group 3: Absence of drop collapse at low respiration rates when exposed to either blue or white LEDs; Group 4: Absence of drop collapse when exposed to either blue or white LEDs with lower respiration for blue and maintained for white LED treatments; Group 5: Enhanced drop collapse when exposed to blue LED, but not to other light regimes). N, nitrogen; P, phosphorus; S, sulfur.*

The short-wave length blue LED regime impaired the drop collapse response. Five dominant patterns related to blue short-wave light spectrum were found (Table 3):

Absence of drop collapse at lower respiration rates when exposed to blue LEDs compared with the other light regimes (Group 1).
Absence of drop collapse when exposed to blue LEDs, but respiration rates comparable between the four light regimes (Group 2).
Absence of drop collapse at low respiration rates when exposed to either blue or white LEDs (Group 3).
Absence of drop collapse when exposed to either blue or white LEDs with lower respiration for blue and maintained for white LED treatments (Group 4).
Enhanced drop collapse when exposed to blue LEDs, but not to other light regimes (Group 5).

The latter group is of specific interest. Of these four compounds, substrate utilization was low (<50 omnilog units) irrespective of light treatment for α-keto-valeric acid, 2-hydroxy-benzoic acid (PA), and γ-hydroxy-butyric acid (PDR5-09), but high for L-arabinose (PA). Light-dependent effects were only found in the case of α-keto-valeric acid, for which utilization under blue LED exposure (average omnilog units: 25.14) was significantly lower than under dark conditions (average omnilog units: 43.83) (*p* < 0.019).

**FIGURE 6:**
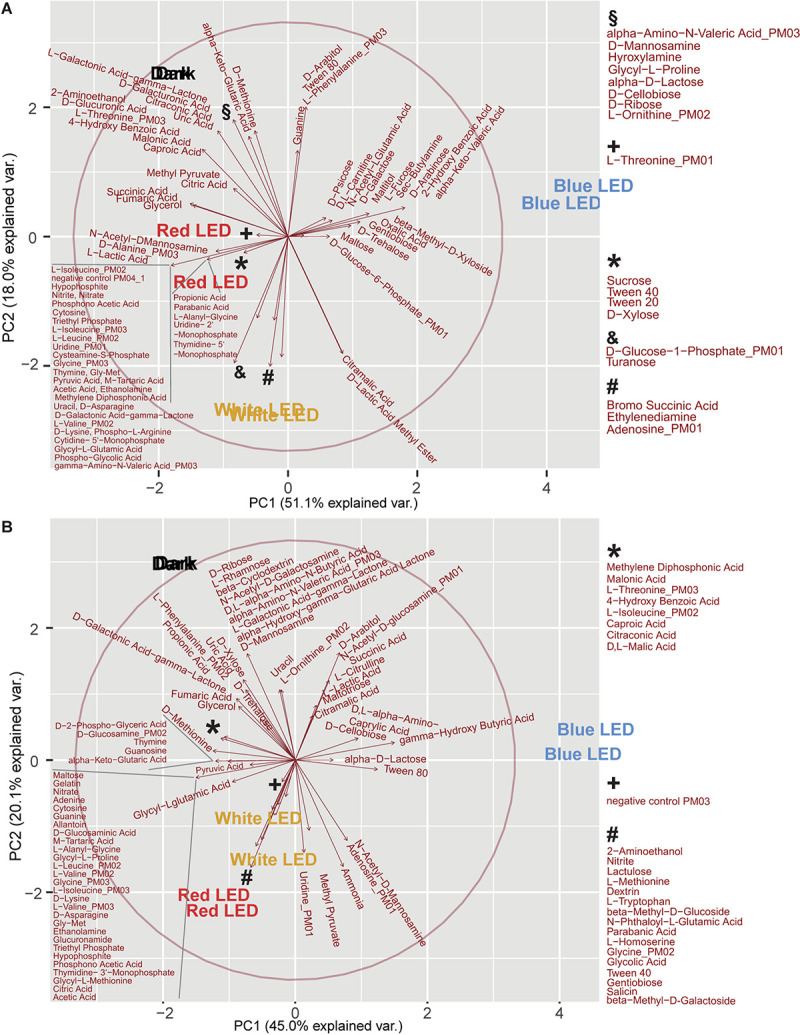
Principal component analysis (score plots, loading plots) of light regime and substrate utilization interactions on biosurfactant formation of *P. agarici* **(A)** and *Pseudomonas* sp. DR 5-09 **(B)**. The analysis was based on two repetitions. These two repetitions display a very similar picture for the scores, therefore the text for the treatments is close to each other or overlapping.

For both of these strains, principal component (PC) 1 discriminated between blue LED exposure and the other three light regimes (PC1: PA: 51.1%, PDR 5-09: 45.0%), while PC2 differentiated between dark incubation and red and white LEDs, respectively (PC2: PA: 18.0%, PDR5-09: 20.1%). For the blue LED regime, D-psicose, D, L-carnitine, *N*-acetyl-L-glutamic acid, D-galactose, malitol, L-fucose, butylamine (sec), D-arabinose, 2-hydroxy benzoic acid, α-keto-valeric acid, β-methyl-D-xyloside, oxalic acid, gentiobiose, D-trehalose, D-glucose-6-phosphate (C source), and maltose (PA) (Figure 6A), and for PDR5-09 arabitol, *N*-acetyl-D-glucosamine (C source), succinic acid, L-citrulline, L-lactic acid, maltotriose, citramalic acid, D,L-α-amino-caprylic acid, D-cellobiose, γ-hydroxy butyric acid, α-D-lactose as well as Tween 80 had the most decisive loads (Figure 6B). Further analysis of the impact of light spectrum on Tween 20, 40, and 80 is presented in Supplementary Appendix A and Supplementary Figure 3.

The importance of inorganic and organic N sources for formation of surface active compounds was reflected in the large number of biosurfactant-positive substrates (33%). Ammonia was intensively used in all light regimes and displayed a positive drop collapse for both strains. Except for the blue LED treatment, this also applied to sodium nitrate and sodium nitrite. Substrate richness was critical for biosurfactant formation, *e.g.*, biosurfactants were exclusively formed by PA when D-alanine, L-phenylalanine, glycine, L-threonine, α-amino-*N*-valeric acid, and γ-amino-*N*-valeric acid were supplied as an N, but not C, source. In contrast, in the presence of either sole or enriched iso-leucine, biosurfactant production was observed, but not after exposure to blue LEDs. Precursor preferences for biosurfactant formation of PDR5-09 were not fully congruent with those of PA. Glycine utilization was found exclusively after red LED treatment when glycine served as a C source, but also after dark incubation and white LED exposure when it served as an N source. L-Threonine and α-amino-*N*-valeric acid amended as N sources supporting biosurfactant formation. Both the sole and enriched form of L-isoleucine supported biosurfactant formation, but only during dark and red LED incubation in the sole state and all others except blue LEDs in the enriched state.

### End Point Biofilm Quantification as a Response to Nutrient Availability and Light Spectrum

**FIGURE 7:**
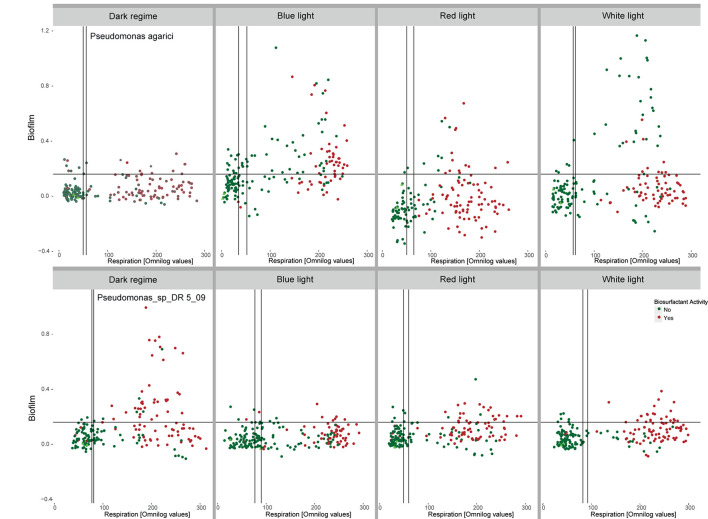
Interactions between light regime (dark incubation, incubation in the presence of short waved blue and long waved red monochromatic LEDs as well as polychromatic white LEDs), respiration of 190 C-sources by *Pseudomonas agarici* and *Pseudomonas* sp. DR5-09 and biofilm formation quantified at incubation endpoint. Green and red dots indicate the absence and presence, respectively, of endpoint biosurfactant activity in the individual wells. The experiment was conducted with two replicate panels using Omnilog phenotypic microarray (PM1 and 2).

The amount of biofilm quantified at the end of the incubation varied in response to supplied nutrients and light spectrum, but also differed between the two *Pseudomonas* strains. When PA was incubated in the absence of light, no increased biofilm formation was stated with increasing respiration, irrespective of endpoint biosurfactant activity in the incubated suspension. In the presence of the three light regimes, C-source respiration and biofilm formation occurred to be correlated. This was most expressed for C-sources exposed to short waved blue LEDs and polychromatic white LEDs. Especially in the presence of white LEDs, the highest amounts of biofilm were formed in the presence of sources where no endpoint biosurfactant activity was detected. Under long waved red LED conditions, a majority of sources with biofilm formation also showed biosurfactant activity (Figure 7). In contrast, PDR5-09 produced biofilm predominantly under dark incubation, but not to a larger extent when irradiated and no interaction was found with biosurfactant activity as evaluated at endpoint.

## Discussion

Recent technological innovations regarding LEDs have led to a shift in supplementary lighting in greenhouse horticulture, by allowing tailored light bands to optimize plant properties (*e.g.*, photosynthetic activity, accumulation of bioactive compounds, plant architecture) (Massa et al., 2008; Morrow, 2008; Demotes-Mainard et al., 2016; Huché-Thélier et al., 2016; Alsanius et al., 2019). Light-microbe interactions in the phyllosphere are an important issue in the altered system, for example with respect to control of plant pathogens and maintaining biological control activity of antagonists. Our findings add novel information on the behavior of epiphytes in different light spectra that is important both for modern horticultural production technology in greenhouses and plant factories. Apart from enabling tailored light spectra, artificial LED lighting technology allows the light spectrum to be modified under field conditions using light-filtering screens. This may be of use for ensuring more consistent success of biocontrol agents when applying them together with relevant precursor compounds, coupled with light regimes that promote the spread of the biocontrol agent in an initial phase and biofilm formation in a secondary phase.

Interactions between phyllosphere microorganisms and visible light spectrum have received only little attention in previous research and then mainly with the focus on the impact on plant pathogenic organisms (Alsanius et al., 2019). However, capacity of non-phototrophic bacteria to sense light has been described for various bacteria and has received attention in medical treatment of skin infections (Dai et al., 2012; Maclean et al., 2014; Wang et al., 2016). Light-sensitive receptor proteins in bacteria are similar to those occurring in plants and LOV-HK, BLUF, and phytochrome have been verified for *P. aeruginosa*, *P. syringae*, *Xanthomonas axnopodis* pv. *citri*, *Rhodopseudomonas pallustris*, and *Bacillus subtilis*, respectively. Bacterial lifestyle changes in response to light have recently been reported. With respect to phyllosphere bacteria, the few studies published to date focus on the plant pathogenic bacterium *P. syringae*. To understand metabolic responses to light spectrum, Gharaie et al. (2017a) recently developed a model system based on the Biolog platform for Phenotype MicroArray. They showed that PDR5-09 distinctly changed its substrate utilization pattern when exposed to blue or white LEDs, as opposed to incubation under dark or red LEDs.

### Light-Dependent Reaction Norms

In the context of zooecological issues, Miner et al. (2005) emphasized the meta-impact of PP, i.e., not only the direct and indirect effects of plasticity in terms of adaptive responses and changes in consumption patterns, but also the impact on the community structure due to phenotypic alterations. The results from the present study indicate that blue light impairs the utilization of sole and enriched substrates by the two selected *Pseudomonas* strains and has similar, but less pronounced, implications for SG, whereas substrate utilization by Bti appears to be indifferent to light regime during incubation (Figures 1, 3, 4).

Bacterial PP has been studied with respect to different plant habitats (soil, rhizosphere, phyllosphere) (Wilson and Lindow, 1993; Germida et al., 1998; Monier and Lindow, 2003) and for stresses relevant for the phyllosphere, e.g., desiccation, but also with respect to different pre-treatments (Wilson and Lindow, 1993; Monier and Lindow, 2003). These studies predominantly investigate *Pseudomonas* strains. Gram-positive bacteria inhabiting plants have not previously been studied with respect to PP. In this study, both SG and Bti utilized the supplied substrates to a much lower extent than the Gram-negative bacteria. The results also indicated that Bti was not affected by different light spectra, whereas SG was affected by light spectrum but did not show a distinct blue light effect. Thus predominantly genetic and to a smaller extent environmental variance, but not GxE interactions, were displayed. In heatmaps, there was a mixed blue/white LED cluster, indicating variable responses for different replicates, despite the robust experimental conditions (Gharaie et al., 2017a).

### Substrate Utilization

Germida et al. (1998) attributed differences in survival of *P. aureofaciens* in the soil and rhizosphere to nutritional effects. However, as they found only small changes in C source utilization and fatty acid methylester patterns were altered marginally, they concluded the *P. aureofaciens* has little phenotypically plasticity and exhibits minor phenotypic drift. Although the *Pseudomonas* strains investigated here showed high PP competences due to light, this ability might not be a general feature of the genus *Pseudomonas*. An interesting candidate for further characterization would thus be *P. aureofaciens*, starting with investigating the genetic makeup regarding light receptor proteins/structures (like ours). Exposure of *P. syringae* to dessication when inoculated to the phyllosphere under different conditions demonstrated that its environmental memory (“pre-adaptation”) is a significant factor for its survival in drought (Wilson et al., 1999). The present investigation is the first to simultaneously study interactions of light spectrum and nutritional factors and to demonstrate the involvement of PP under blue light conditions. All strains were cultured for a longer time after isolation and pre-adaptation can be excluded as a reason for differences in performance. In this study and in all similar previous investigations, reversibility/irreversibility in the phenotypic response has been considered and is interesting for understanding the consequences of PP. There are indications from studies of LOV proteins originating from *P. putida* that the reversibility of blue light effects and recovery kinetics are LOV protein-dependent (Krauss, 2008).

**FIGURE 8:**
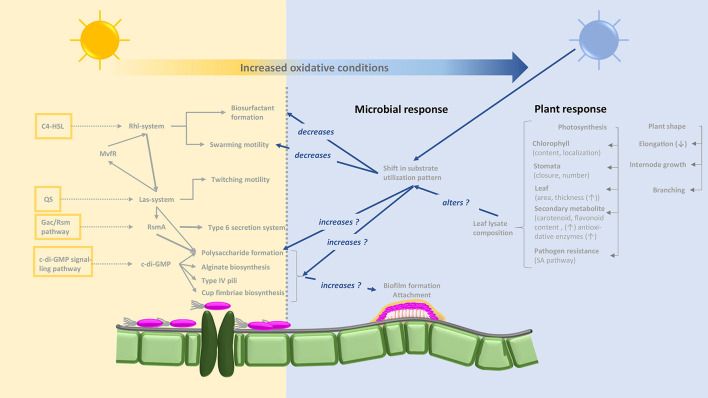
Concept-based map illustrating putative interactions between light regime (non-blue light: yellow background; blue light: blue background), plant responses and responses of *Pseudomonas* sp. based on the results obtained and including literature knowledge. Pathways for biosurfactant and biofilm formation are based on Kyoto Encyclopedia of Genes and Genomes (KEGG) map02025 “Biofilm formation–Pseudomonas aeruginosa” (http://www.kegg.jp/kegg-bin/highlight_pathway?scale=1.0&map=map02025&keyword=biofilmPseudomonas). The yellow framed boxes on the left display pathways involved into biofilm formation and the dotted arrows link to the main signaling cascades. The C4-HSL = *N*-butyryl-l-homoserine lactone, links to the Rhl-system = two lux-type quorum sensing systems, and further to the Las-system. In *Pseudomonas aeruginosa* this is consisting of two components, namely an autoinducer synthesizing acyl homoserine lactone (AHL) from methionine and a transcriptional controller and mediates the formation of C4-HSL (*N*-butanoyl homoserine) which enables communication between bacterial cells among others biofilm formation. Interlinked between these the MvfR = transcriptional regulator moderating the expression of QS-based virulence factors, mediates a feedback system. The QS, quorum sensing, also acts into the Las-system impacting together with the Gac, global regulatory system for activation of antibiotic and cyanide synthesis, the RmsA, small RNA binding protein; the Gac/Rms pathway allows the organism to switch between primary and secondary metabolism; c-di-GMP, Bis-(3′–5′)-cyclic dimeric guanosine monophosphate, then also majorly acts on processes of the biofilm formation *via* impacting features like Polysaccharide formation, alginate biosynthesis, etc. According to the experimental findings, it is clear, that blue light regimes impacts several features of bacterial habit in the indicated way. However, it needs further investigation to identify the molecular mechanisms and locations of these influences.

Due to the absence of blue light receptor proteins in the two Gram-positive strains tested here, it is tempting to ascribe the effects observed to the putative blue light receptor proteins retrieved from the genomic blasting of the two *Pseudomonas* strains (Figure 2). Non-phototrophic bacteria, but also plants, can be affected by blue light. As a general phenomenon, oxidative stress increases under blue light conditions (Figure 8). Plants display both morphological (plant shape, branching, decreased elongation, leaf area, and increased leaf thickness) and physiological (chlorophyll content and location, stomata number and closure, species-dependent increased secondary metabolite formation, and induction of the immune defense system) responses to longer exposure to blue light (Huché-Thélier et al., 2016; Taulavuori et al., 2016). Putative blue light receptor domains (LOV) have been identified in various *Pseudomonas* species (*P. aeruginosa*, *P. entomophila*, *P. fluorescens*, *P. mendocina*, *P. putida*, and *P. syringae*) (van der Horst et al., 2007) and lifestyle changes from a planktonic to a biofilm-associated lifestyle when exposed to blue light have been reported previously for both clinical and plant-associated strains (Wu et al., 2013; Río-Álvarez et al., 2014). This phenomenon may be viewed as protection against environmental, but also plant-induced, oxidative stress. Decreased surfactant formation in the presence of certain substrates when exposed to blue light appears to be a logical assumption in this context (Figure 8). On the basis of altered substrate utilization by *Pseudomonas* sp., our data indicate the induction of physiological switches as a result of blue light exposure. Although some deviations occurred between the two strains with respect to their respiration of different sole and enriched substrates under different light conditions, the grouping of utilization patterns depending on the light source is robust. The nutritional dimension of such switches has been shown for Fe-deficient conditions in LOV gene clusters cloned from *P. putida*, which promote siderophore production (Krauss, 2008). Our data from two different *Pseudomonas* species emphasize the importance of nutritional conditions for light spectrum experiments and thus complement the two previous investigations.

### Light-Dependent Lifestyle Changes and Habitat Modifications

The formation of biosurfactant by *Pseudomonas* in the presence of the substrates tested was affected by the short-wave light spectrum, indicating that changes in the distribution on the leaf surface and, hence, of the community structure might also be expected. These interactions have not been reported previously for any of the chosen model organisms. They support our hypothesis that the light spectrum affects the PP of epiphytic phyllosphere colonizers, but also indicate that this applies to certain, but definitely not all, epiphytic bacteria (Figures 3, 6 and Table 3).

Various compounds have been depicted as precursor substrates for biosurfactant formation by *Pseudomonas* strains, including the carbohydrates glucose (Guerra-Santos et al., 1984; Dubern and Bloemberg, 2006), glycerol (Rahman et al., 2002; Dubern and Bloemberg, 2006; Silva et al., 2010), succinic acid (Dubern and Bloemberg, 2006), sucrose (Persson and Molin, 1987; Dubern and Bloemberg, 2006), and the amino acids L-threonine, L-valine, L-leucine, L-isoleucine, and L-serine (Dubern and Bloemberg, 2006). These substrates are also part of the panels used in the present study. However, biosurfactant formation could not be confirmed for all of these compounds under dark conditions (none of the strains: sucrose, L-threonine, L-serine). Lipid-rich, but not carbohydrate-rich, material is important for rhamnolipid formation (Nitschke et al., 2005). The chemical nature of the active compound behind the surface activity of the strains selected in this study has not yet been characterized, and therefore, it is difficult to draw conclusions on substrate preferences with respect to surfactant formation. Several patterns of precursor respiration rates and biosurfactant formation have been found. High substrate utilization rates linked to surfactant formation may be explained by literature findings on biosurfactant formation during the stationary phase (Figure 3). This also explains the absence or inconsistent formation of biosurfactants by the two Gram-positive strains. Dubern et al. (2006) proposed a causal interaction between cell density, induction of quorum-sensing signals and production of the surface active lipopeptide putisolvin. The significance of abiotic factors, such as salt concentration, nutrient relationships, temperature, pH and oxygen concentration, has been reported previously (Guerra-Santos et al., 1984; Rahman and Gakpe, 2001; Dubern and Bloemberg, 2006; Silva et al., 2010). Our findings are novel in demonstrating the impact of different light spectra and light regimes. Based on these findings, further studies are needed on cell density assessment and transcriptomic analysis of whether blue LED treatment causes a metabolic switch or barely slows down the generation time and metabolite formation (Figure 8). The exclusive capacity of PA and PDR5-09 to stimulate consistent biosurfactant formation in the presence of short-wave blue and four distinct C sources (Table 2, group 5) is an interesting and novel finding that needs further investigation on pathways, general incidence, and importance for plant-associated contexts.

As different nutrient components may be required to optimize the yield of different surfactant agents, no general conclusions can be drawn from the present study regarding specific surfactants. In a crop ecology context, biosurfactants play a critical role in affecting certain plant pathogenic structures (zoospore lysis) (Raaijmakers et al., 2006; Hultberg et al., 2010a,b; Raaijmakers et al., 2010) and in exploring and conquering new habitats on the plant surface. Leaf cuticles covered with a waxy layer are hydrophobic surfaces. The ability to lower surface tension, but also to disrupt existing biofilms, is therefore an important property for microbes seeking new habitats on the leaf surface or once established, to defend against invading foreign species. Our results indicated that the ability to explore new habitats on the leaf surface is a function of both light spectrum and presence of certain precursor compounds. This is the first study in which biosurfactant formation was combined with the Biolog phenotypic microarray platform. Little is known about the nutritional situation on the leaf surfaces, but sole resource situations are most likely absent. Previous reports of sole sugar compounds dominating in the phyllosphere (Mercier and Lindow, 2000; Leveau and Lindow, 2001; Aruscavage et al., 2010) appear to be of little relevance in the present case. Our results indicated a detrimental effect of some co-catabolically used substrates on biosurfactant formation, especially under blue LED conditions, which supports our hypothesis that the bacterial response to nutritional and light conditions is reflected in the strain’s capacity to form biosurfactants. This finding is in line with observations of decreased swarming motility and increased biofilm formation by *Pseudomonas syringae* pv. *tomato* (Río-Álvarez et al., 2014). These insights can open up new approaches for more successful establishment of *Pseudomonas* biocontrol strains by altering motility and biofilm formation after inoculation by means of light spectrum strategies. Conversely, it may be possible to counteract plant pathogenic bacteria through light treatment.

Both biosurfactant and biofilm formation are important mechanisms for establishment of phyllosphere bacteria. The interdependence between nutrient availability and biofilm formation is a well-known fact (see Alsanius et al., 2019 and references therein). However, less light has been shed on interactions between light spectrum and biofilm formation on terrestrial plant surfaces and non-phototrophic bacteria (Yu and Lee, 2013). It is tempting to translate the present findings into global conclusions. However, the present results do not elucidate the causes behind biofilm formation. If these effects are a result of the light source exclusively or of nutrient depletion cannot be determined from the present dataset. Likewise, the interesting finding of simultaneous endpoint biosurfactant activity and biofilm formation in the presence of high respiration raises the question about chronology of events, requiring kinetic studies as well as transcriptome analysis under steady state and spiked conditions.

To conclude, our results show that:

Phenotypic plasticity occurs in non-phototrophic bacteria in response to light spectrum exposure, but is not a universal characteristic.
Blue LED light leads to considerable changes in the metabolism of *Pseudomonas* strains and,
Blue LED light alters the capacity to form biosurfactants.
Blue and white LEDs can alter the presence of biofilm at endpoint in certain Pseudomonas species.
Further studies need to elucidate the sequential arrangement of metabolite formation and their dependence of number and position of blue light receptor domains.

## Data Availability Statement

The datasets generated during and analyzed during the current study are available in the https://hdl.handle.net/20.500.12703/3896 repository.

## Author Contributions

BA and SW: conceptualization and methodology. LV and BA: validation, formal analysis, writing—original draft preparation, and visualization. SG, AR, MK, and BA: investigation. BA: resources, project administration, and funding acquisition. LV, BA, SG, AR, and MK: data curation. BA, SW, LV, SG, AR, MK, WW, and SK: writing—review and editing. BA, SK, WW, and SW: supervision.

## Conflict of Interest

The authors declare that the research was conducted in the absence of any commercial or financial relationships that could be construed as a potential conflict of interest.

## Publisher’s Note

All claims expressed in this article are solely those of the authors and do not necessarily represent those of their affiliated organizations, or those of the publisher, the editors and the reviewers. Any product that may be evaluated in this article, or claim that may be made by its manufacturer, is not guaranteed or endorsed by the publisher.

## Footnotes

1
http://blast.ncbi.nlm.nih.gov/Blast.cgi

2
http://folk.uio.no/ohammer/past/

3
http://www.genome.jp/kegg/

## Supplementary Material

Supplementary Figure 2 Experimental set-up for screening for biosurfactant formation and ranking scheme for drop collapse (0–3; with 0 = convex droplet, no biosurfactant formation; 1 = moderately convex droplet, moderate biosurfactant formation; 2 = flattened droplet, biosurfactant formation) (illustration: BA).

Supplementary Table 1 Substrate overlay on the four selected PM panels (PM1, PM2, PM3, and PM4 according to Biolog Inc., Haywood, United States).

Supplementary Table 3 Substrate names from Figure 4. Substrate names are listed bottomup, as they occured as the Y-axis labels in the respective heatmap.

Supplementary Appendix A Interactions between *Pseudomonas agarici* and *Pseudomonas* sp. DR 5-09 and biosurfactant activity when incubated in Tween 20, Tween 40, and Tween 80 and different light qualities. Supplementary Appendix A includes Supplementary Figure 4.

## Abbreviations

BLAST: basic local alignment search tool
PAS: domain Per-Arnt-Sim
PDR5-09: *Pseudomonas* sp. DR 5-09
PA: *Pseudomonas agarici*
Bti: *Bacillus thuringiensis* serovar *israelensis*
SG: *Streptomyces griseoviridis*
PP: phenotypic plasticity.
