# Supplementary material for: Dining in Blue Light Impairs the Appetite of Some Leaf Epiphytes
Source: Front Microbiol. 2021 Oct 18;12:725021. doi: 10.3389/fmicb.2021.725021 (PMC8558677; doi:10.3389/fmicb.2021.725021)
Supplement: Supplementary Figure 2 — Experimental set-up for screening for biosurfactant formation and ranking scheme for drop collapse (0–3; with 0 = convex droplet, no biosurfactant formation; 1 = moderately convex droplet, moderate biosurfactant formation; 2 = flattened droplet, biosurfactant formation) (illustration: BA). [file Data_Sheet_1.zip › Apendix A and Figure S4.pdf]

Appendix A.

Interactions between Pseudomonas agarici and Pseudomonas sp. DR 5-09 and biosurfactant activity when incubated in Tween 20, Tween 40 and Tween 80 and different light qualities

Tween 20, Tween 40 and Tween 80 are themselves surfactants and a positive reaction in the drop collapse test is therefore not surprising. However, the four test strains utilised these three compounds differently and light spectrum-dependent variations were found for Tween 20, Tween 40 and Tween 80 in *P. agarici* and Tween 80 in *Pseudomonas* sp. DR 5-09 (Figure S4). They were therefore not excluded from the data analysis. Drop collapse for the three surfactant compounds after incubation was also a function of light regime. For example, dark-incubated and white and red LED-exposed wells of Tween 80 incubated with *P. agarici* displayed high surface activity, but not blue LED-exposed wells. Likewise, only suspensions from red LED-exposed wells of Tween 20 and Tween 40 showed surface activity. These results were not matched by the assay with *Pseudomonas* sp. DR 5-09; surface activity of Tween 80 suspensions remained in blue LED-exposed wells and was inconsistent in white LED-exposed wells, but was absent for any of the light regimes in Tween 20 and Tween 40 suspensions. No consistent pattern of interactions between substrate respiration and surface activity was found.

Interestingly, different responses to light regime were found on incubating the two *Pseudomonas* strains in the surfactants Tween 20, Tween 40 and Tween 80. A blue light regime limited respiration of the different configurations of Tween, especially for *Pseudomonas* sp. DR 5-09. A negative correlation between respiration rate and intensity of surface activity, as stated for Tween 20 at all light regimes, might indicate capacity to be utilised as a carbon source. Maintained high surface activity associated with high respiration, as displayed by *P. agarici* for Tween 40 under red LED, might either indicate an additive effect or the formation of another surface active metabolite while Tween 40 is utilised as a C source. Low respiration of Tween combined with surface activity in the drop collapse test (*Pseudomonas* sp. DR 5-09: Tween 80/blue LED) might indicate lack of capacity to utilise the compound under those conditions, hence the unaffected surface activity of the substrate.

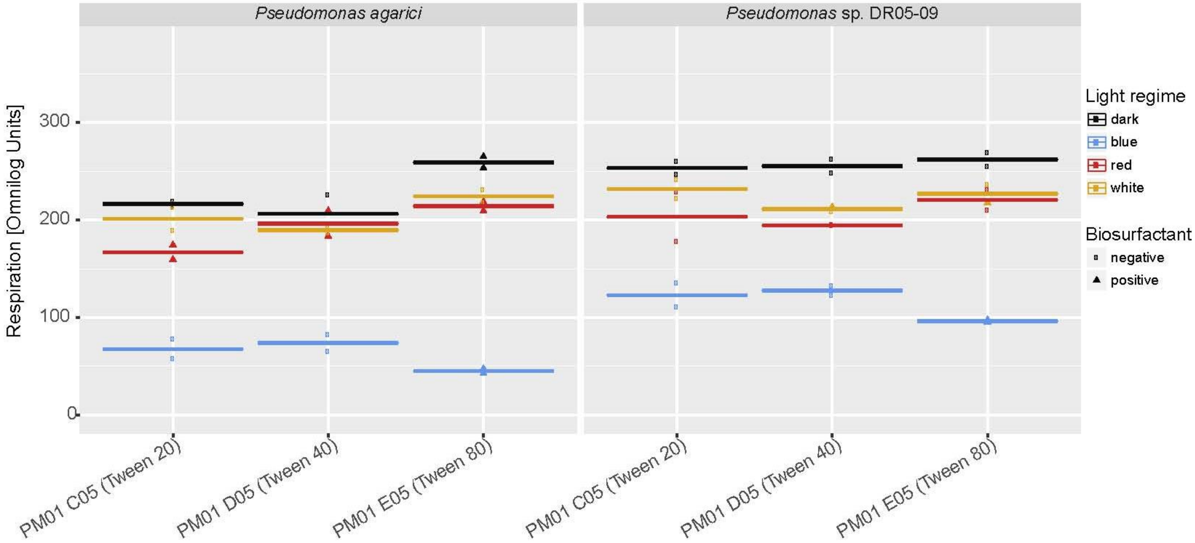

Supplementary Figure S4. Impact of light regime on utilisation and surface activity of Tween 20, Tween 40 and Tween 80 by *Pseudomonas agarici* and *Pseudomonas* sp. DR 5-09.
