# Supplementary material for: Dining in Blue Light Impairs the Appetite of Some Leaf Epiphytes
Source: Front Microbiol. 2021 Oct 18;12:725021. doi: 10.3389/fmicb.2021.725021 (PMC8558677; doi:10.3389/fmicb.2021.725021)
Supplement: Supplementary Figure 2 — Experimental set-up for screening for biosurfactant formation and ranking scheme for drop collapse (0–3; with 0 = convex droplet, no biosurfactant formation; 1 = moderately convex droplet, moderate biosurfactant formation; 2 = flattened droplet, biosurfactant formation) (illustration: BA). [file Data_Sheet_1.zip › Figure S2.PDF]

Supplement figure S2

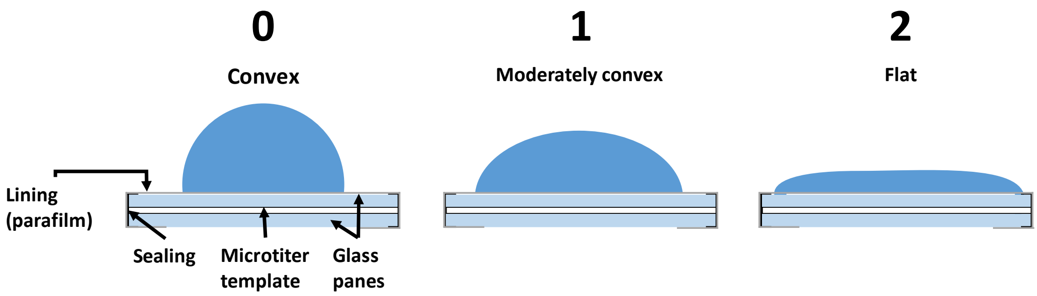

Supplement Figure S2. Experimental set-up for screening for biosurfactant formation and ranking scheme for drop collapse (0-3; with 0 = convex droplet, no biosurfactant formation; 1 = moderately convex droplet, moderate biosurfactant formation; 2 = flattened droplet, biosurfactant formation) (illustration: B. Alsanius)
