# Supplementary material for: Dining in Blue Light Impairs the Appetite of Some Leaf Epiphytes
Source: Front Microbiol. 2021 Oct 18;12:725021. doi: 10.3389/fmicb.2021.725021 (PMC8558677; doi:10.3389/fmicb.2021.725021)
Supplement: Supplementary Figure 2 — Experimental set-up for screening for biosurfactant formation and ranking scheme for drop collapse (0–3; with 0 = convex droplet, no biosurfactant formation; 1 = moderately convex droplet, moderate biosurfactant formation; 2 = flattened droplet, biosurfactant formation) (illustration: BA). [file Data_Sheet_1.zip › Table S1.PDF]

Suppl Table S1. Substrate overlay on the four selected PM panels (PM1, PM2, PM3, PM4 according to Biolog Inc., Haywood, USA)

Position	PM01	PM02	PM03	PM04
	Carbon sources		*Nitrogen sources*	*Phosphorus and sulphur sources*
A01	Negative Control	Negative Control	Negative Control	Negative Control
A02	L-Arabinose	Chondroitin Sulfate C	Ammonia	Phosphate
A03	N-Acetyl-D-Glucosamine	α-Cyclodextrin	Nitrite	Pyrophosphate
A04	D-Saccharic Acid	β-Cyclodextrin	Nitrate	Trimeta-phosphate
A05	Succinic Acid	γ-Cyclodextrin	Urea	Tripoly-phosphate
A06	D-Galactose	Dextrin	Biuret	Triethyl Phosphate
A07	L-Aspartic Acid	Gelatin	L-Alanine	Hypophosphite
A08	L-Proline	Glycogen	L-Arginine	Adenosine- 2′-monophosphate
A09	D-Alanine	Inulin	L-Asparagine	Adenosine- 3′-monophosphate
A10	D-Trehalose	Laminarin	L-Aspartic Acid	Adenosine- 5′-monophosphate
A11	D-Mannose	Mannan	L-Cysteine	Adenosine- 2′,3′-cyclic monophosphate
A12	Dulcitol	Pectin	L-Glutamic Acid	Adenosine- 3′,5′-cyclic monophosphate
B01	D-Serine	N-Acetyl-D-Galactosamine	L-Glutamine	Thio-phosphate
B02	D-Sorbitol	N-Acetyl-Neuraminic Acid	Glycine	Dithio-phosphate
B03	Glycerol	β-D-Allose	L-Histidine	D,L-α-Glycerol Phosphate
B04	L-Fucose	Amygdalin	L-Isoleucine	β-Glycerol Phosphate
B05	D-Glucuronic Acid	D-Arabinose	L-Leucine	Carbamyl Phosphate
B06	D-Gluconic Acid	D-Arabitol	L-Lysine	D-2-Phospho-Glyceric Acid
B07	D,L-α-Glycerol- Phosphate	L-Arabitol	L-Methionine	D-3-Phospho-Glyceric Acid
B08	D-Xylose	Arbutin	L-Phenylalanine	Guanosine- 2′-monophosphate
B09	L-Lactic Acid	2-Deoxy-D-Ribose	L-Proline	Guanosine- 3′-monophosphate
B10	Formic Acid	i-Erythritol	L-Serine	Guanosine- 5′-monophosphate
B11	D-Mannitol	D-Fucose	L-Threonine	Guanosine- 2′,3′-cyclic monophosphate
B12	L-Glutamic Acid	3-0-β-D-Galacto-pyranosyl-D-Arabinose	L-Tryptophan	Guanosine- 3′,5′-cyclic monophosphate
C01	D-Glucose-6-Phosphate	Gentiobiose	L-Tyrosine	Phosphoenol Pyruvate
C02	D-Galactonic Acid-γ-Lactone	L-Glucose	L-Valine	Phospho- Glycolic Acid
C03	D,L-Malic Acid	Lactitol	D-Alanine	D-Glucose-1-Phosphate
C04	D-Ribose	D-Melezitose	D-Asparagine	D-Glucose-6-Phosphate
C05	Tween 20	Maltitol	D-Aspartic Acid	2-Deoxy-D-Glucose 6-Phosphate
C06	L-Rhamnose	α-Methyl-D-Glucoside	D-Glutamic Acid	D-Glucos-amine-6-Phosphate
C07	D-Fructose	β-Methyl-D-Galactoside	D-Lysine	6-Phospho-Gluconic Acid
C08	Acetic Acid	3-Methyl Glucose	D-Serine	Cytidine- 2′- monophosphate
C09	α-D-Glucose	β-Methyl-D-Glucuronic Acid	D-Valine	Cytidine- 3′- monophosphate
C10	Maltose	α-Methyl-D-Mannoside	L-Citrulline	Cytidine- 5′- monophosphate
C11	D-Melibiose	β-Methyl-D-Xyloside	L-Homoserine	Cytidine- 2′,3′-cyclic monophosphate
C12	Thymidine	Palatinose	L-Ornithine	Cytidine- 3′,5′-cyclic monophosphate
D01	L-Asparagine	D-Raffinose	N-Acetyl-L-Glutamic Acid	D-Mannose-1-Phosphate
D02	D-Aspartic Acid	Salicin	N-Phthaloyl-L-Glutamic Acid	D-Mannose-6-Phosphate
D03	D-Glucosaminic Acid	Sedoheptulosan	L-Pyroglutamic Acid	Cysteamine-S-Phosphate
D04	1,2-Propanediol	L-Sorbose	Hydroxylamine	Phospho-L-Arginine
D05	Tween 40	Stachyose	Methylamine	O-Phospho-D-Serine
D06	α-Keto-Glutaric Acid	D-Tagatose	N-Amylamine	O-Phospho-L-Serine
D07	α-Keto-Butyric Acid	Turanose	N-Butylamine	O-Phospho-L-Threonine
D08	α-Methyl-D-Galactoside	Xylitol	Ethylamine	Uridine- 2′- monophosphate
D09	α-D-Lactose	N-Acetyl-D-Glucosaminitol	Ethanolamine	Uridine- 3′- monophosphate
D10	Lactulose	γ-Amino Butyric Acid	Ethylenediamine	Uridine- 5′- monophosphate
D11	Sucrose	δ-Amino Valeric Acid	Putrescine	Uridine- 2′,3′- cyclic monophosphate
D12	Uridine	Butyric Acid	Agmatine	Uridine- 3′,5′- cyclic monophosphate
E01	L-Glutamine	Capric Acid	Histamine	O-Phospho-D-Tyrosine
E02	m-Tartaric Acid	Caproic Acid	β-Phenylethyl- amine	O-Phospho-L-Tyrosine
E03	D-Glucose-1-Phosphate	Citraconic Acid	Tyramine	Phospho-creatine
E04	D-Fructose-6-Phosphate	Citramalic Acid	Acetamide	Phosphoryl Choline
E05	Tween 80	D-Glucosamine	Formamide	O-Phosphoryl-Ethanolamine
E06	α-Hydroxy Glutaric Acid-γ-Lactone	2-Hydroxy Benzoic Acid	Glucuronamide	Phosphono Acetic Acid
E07	α-Hydroxy Butyric Acid	4-Hydroxy Benzoic Acid	D,L-Lactamide	2-Aminoethyl Phosphonic Acid
E08	β-Methyl-D-Glucoside	β-Hydroxy Butyric Acid	D-Glucosamine	Methylene Diphosphonic Acid
E09	Adonitol	γ-Hydroxy Butyric Acid	D-Galactosamine	Thymidine- 3′-monophosphate
E10	Maltotriose	α-Keto-Valeric Acid	D-Mannosamine	Thymidine- 5′-monophosphate
E11	2-Deoxy Adenosine	Itaconic Acid	N-Acetyl-D-Glucosamine	Inositol Hexa-phosphate
E12	Adenosine	5-Keto-D-Gluconic Acid	N-Acetyl-D-Galactosamine	Thymidine 3′,5′- cyclic mono-phosphate
F01	Glycyl-L-Aspartic Acid	D-Lactic Acid Methyl Ester	N-Acetyl-D-Mannosamine	Negative Control
F02	Citric Acid	Malonic Acid	Adenine	Sulfate
F03	m-Inositol	Melibionic Acid	Adenosine	Thiosulfate
F04	D-Threonine	Oxalic Acid	Cytidine	Tetrathionate
F05	Fumaric Acid	Oxalomalic Acid	Cytosine	Thio-phosphate
F06	Bromo Succinic Acid	Quinic Acid	Guanine	Dithio-phosphate
F07	Propionic Acid	D-Ribono-1,4-Lactone	Guanosine	L-Cysteine
F08	Mucic Acid	Sebacic Acid	Thymine	D-Cysteine
F09	Glycolic Acid	Sorbic Acid	Thymidine	L-Cysteinyl-Glycine
F10	Glyoxylic Acid	Succinamic Acid	Uracil	L-Cysteic Acid
F11	D-Cellobiose	D-Tartaric Acid	Uridine	Cysteamine
F12	Inosine	L-Tartaric Acid	Inosine	L-Cysteine Sulfinic Acid
G01	Glycyl-L-Glutamic Acid	Acetamide	Xanthine	N-Acetyl-L-Cysteine
G02	Tricarballylic Acid	L-Alaninamide	Xanthosine	S-Methyl-L-Cysteine
G03	L-Serine	N-Acetyl-L-Glutamic Acid	Uric Acid	Cystathionine
G04	L-Threonine	L-Arginine	Alloxan	Lanthionine
G05	L-Alanine	Glycine	Allantoin	Glutathione
G06	L-Alanyl-Glycine	L-Histidine	Parabanic Acid	D,L-Ethionine
G07	Acetoacetic Acid	L-Homoserine	D,L-α-Amino-N-Butyric Acid	L-Methionine
G08	N-Acetyl-β-D-Mannosamine	Hydroxy-L-Proline	γ-Amino-N-Butyric Acid	D-Methionine
G09	Mono Methyl Succinate	L-Isoleucine	ε-Amino-N-Caproic Acid	Glycyl-L-Methionine
G10	Methyl Pyruvate	L-Leucine	D,L-α-Amino- Caprylic Acid	N-Acetyl-D,L-Methionine
G11	D-Malic Acid	L-Lysine	δ-Amino-N-Valeric Acid	L- Methionine Sulfoxide
G12	L-Malic Acid	L-Methionine	α-Amino-N-Valeric Acid	L-Methionine Sulfone
H01	Glycyl-L-Proline	L-Ornithine	Ala-Asp	L-Djenkolic Acid
H02	p-Hydroxy Phenyl Acetic Acid	L-Phenylalanine	Ala-Gln	Thiourea
H03	m-Hydroxy Phenyl Acetic Acid	L-Pyroglutamic Acid	Ala-Glu	1-Thio-β-D-Glucose
H04	Tyramine	L-Valine	Ala-Gly	D,L-Lipoamide
H05	D-Psicose	D,L-Carnitine	Ala-His	Taurocholic Acid
H06	L-Lyxose	Sec-Butylamine	Ala-Leu	Taurine
H07	Glucuronamide	D.L-Octopamine	Ala-Thr	Hypotaurine
H08	Pyruvic Acid	Putrescine	Gly-Asn	p-Amino Benzene Sulfonic Acid
H09	L-Galactonic Acid-γ-Lactone	Dihydroxy Acetone	Gly-Gln	Butane Sulfonic Acid
H10	D-Galacturonic Acid	2,3-Butanediol	Gly-Glu	2-Hydroxyethane Sulfonic Acid
H11	Phenylethylamine	2,3-Butanone	Gly-Met	Methane Sulfonic Acid
H12	2-Aminoethanol	3-Hydroxy 2-Butanone	Met-Ala	Tetra-methylene Sulfone
