# Supplementary material for: Dining in Blue Light Impairs the Appetite of Some Leaf Epiphytes
Source: Front Microbiol. 2021 Oct 18;12:725021. doi: 10.3389/fmicb.2021.725021 (PMC8558677; doi:10.3389/fmicb.2021.725021)
Supplement: Supplementary Figure 2 — Experimental set-up for screening for biosurfactant formation and ranking scheme for drop collapse (0–3; with 0 = convex droplet, no biosurfactant formation; 1 = moderately convex droplet, moderate biosurfactant formation; 2 = flattened droplet, biosurfactant formation) (illustration: BA). [file Data_Sheet_1.zip › Table S3.pdf]

Supplementary Table S3

Substrate names from Figure 5. Substrate names are listed bottomup, as they occured as the y-axis labels in the respective heatmap.

**Subfigure A – PM1 PM2 Carbon sources**	**Subfigure B - PM3 nitrogen sources**	**Subfigure C - PM4 Sulfur sources**	**Subfigure D - PM4 Phosphorus sources**
B12 (L−Glutamic Acid)	A05 (Urea)	G07 (L−Methionine)	
A08 (L−Proline)	A07 (L−Alanine)	G08 (D−Methionine)	C07 (6−Phospho−Gluconic Acid)
C09 (D−Glucose)	B03 (L−Histidine)	F06 (Dithiophosphate #2)	B07 (D−3−Phospho−Glyceric Acid)
F02 (Citric Acid)	B01 (L−Glutamine)	G09 (Gly−Met)	B06 (D−2−Phospho−Glyceric Acid)
D01 (L−Asparagine)	A02 (Ammonia)	F02 (Sulfate)	C06 (D−Glucosamine−6−Phosphate)
B06 (D−Gluconic Acid)	H07 (Ala−Thr)	F07 (L−Cysteine)	C04 (D−Glucose−6−Phosphate)
G12 (L−Malic Acid)	H05 (Ala−His)	G11 (L−Methionine Sulfoxide)	E01 (O−Phospho−D−Tyrosine)
C03 (D,L−Malic Acid)	H06 (Ala−Leu)	G02 (S−Methyl−L−Cysteine)	B04 (b−Glycerol Phosphate)
A02 (L−Arabinose)	F03 (Adenosine)	F12 (L−Cysteine Sulfinic Acid)	C01 (Phosphoenol Pyruvate)
E01 (L−Glutamine)	F02 (Adenine)	F09 (Cys−Gly)	C05
G03 (L−Serine)	A09 (L−Asparagine)	H07 (Hypotaurine)	(2−Deoxy−D−Glucose−6−Phosphate)
H08 (Pyruvic Acid)	A10 (L−Aspartic Acid)	H06 (Taurine)	D05 (O−Phospho−D−Serine)
A03 (N−Acetyl−D−Glucosamine)	B09 (L−Proline)	H11 (Methane Sulfonic Acid)	D06 (O−Phospho−L−Serine)
H03 (L−Pyroglutamic Acid)	B10 (L−Serine)	H10 (2−Hydroxyethane Sulfonic	D04 (Phospho−L−Arginine)
E05 (Tween 80)	A08 (L−Arginine)	Acid)	E04 (Phosphorylcholine)
A07 (L−Aspartic Acid)	A12 (L−Glutamic Acid)	H01 (L−Djenkolic Acid)	E07 (2−Aminoethyl Phosphonic Acid)
A04 (D−Saccharic Acid)	D12 (Agmatine)	H09 (Butane Sulfonic Acid)	A05 (Tripolyphosphate)
F08 (Mucic Acid)	H01 (Ala−Asp)	G04 (Lanthionine)	A02 (Sodium Phosphate)
G08 (L−Hydroxyproline)	D11 (Putrescine)	G10 (N−Acetyl−D,L−Methionine)	A03 (Sodium Pyrophosphate)
A06 (D−Galactose)	H02 (Ala−Gln)	F05 (Thiophosphate #2)	B02 (Dithiophosphate #1)
A05 (Succinic Acid)	H03 (Ala−Glu)	F04 (Tetrathionate)	A08 (Adenosine−2'−Monophosphate)
H08 (Putrescine)	H12 (Met−Ala)	G03 (Cystathionine)	C02 (Phospho−Glycolic Acid)
A11 (D−Mannose)	H08 (Gly−Asn)	F08 (D−Cysteine)	B08 (Guanosine−2'−Monophosphate)
C08 (Acetic Acid)	H09 (Gly−Gln)	F03 (Sodium Thiosulfate)	B05 (Carbamyl Phosphate)
D03 (D−Glucosaminic Acid)	D03 (L−Pyroglutamic Acid)	H05 (Taurocholic Acid)	C08 (Cytidine−2'−Monophosphate)
D05 (Tween 40)	G05 (Allantoin)	H03 (1−Thio−b−D−Glucose)	B03 (D,L−a−Glycerol−Phosphate)
F05 (Fumaric Acid)	G02 (Xanthosine)	H08 (p−Amino Benzene Sulfonic	C03 (a−D−Glucose−1−Phosphate)
B11 (D−Mannitol)	F07 (Guanosine)	Acid)	D02 (D−Mannose−6−Phosphate)
D10 (g−Amino−n−Butyric Acid)	G01 (Xanthine)	G05 (Glutathione)	E12 (Thymidine 3',5'−Cyclic
G06 (Ala−Gly)	F12 (Inosine)	G06 (D,L−Ethionine)	Monophosphate)
G05 (L−Alanine)	E01 (Histamine)	F10 (L−Cysteic Acid)	D07 (O−Phospho−L−Threonine)
E05 (D−Glucosamine)	F06 (Guanine)	G01 (N−Acetyl−L−Cysteine)	

Subfigure A – PM1 PM2 Carbon sources	Subfigure B - PM3 nitrogen sources	Subfigure C - PM4 Sulfur sources	Subfigure D - PM4 Phosphorus sources
B08 (D−Xylose)<br>C04 (D−Ribose)<br>E02 (Caproic Acid)<br>E08 (b−Hydroxy−Butyric Acid)<br>H04 (L−Valine)<br>H12 (Ethanolamine)<br>A09 (D−Alanine)<br>D06 (a−Keto−Glutaric Acid)<br>E02 (m−Tartaric Acid)<br>E03 (Citraconic Acid)<br>B06 (D−Arabitol)<br>F07 (Propionic Acid)<br>F06 (Quinic Acid)<br>G04 (L−Arginine)<br>C05 (Tween 20)<br>G03 (N−Acetyl−L−Glutamic Acid)<br>F01 (D−Lactic Acid Methyl Ester)<br>G09 (L−Isoleucine)<br>E12 (Adenosine)<br>G10 (Methyl Pyruvate)<br>D12 (Uridine)<br>B09 (L−Lactic Acid)<br>F12 (Inosine)<br>C07 (D−Fructose)<br>G01 (Gly−Glu)<br>H02 (L−Phenylalanine)<br>F06 (Bromo−Succinic Acid)<br>C02 (D−Galactonic Acid−g−Lactone)<br>B05 (D−Glucuronic Acid)<br>H10 (D−Galacturonic Acid)<br>H01 (L−Ornithine)<br>G10 (L−Leucine)<br>H09 (L−Galactonic Acid−g−Lactone)<br>F02 (Malonic Acid)<br>E04 (D−Citramalic Acid)<br>H05 (D,L−Carnitine)	C03 (D−Alanine)<br>B04 (L−Isoleucine)<br>C02 (L−Valine)<br>A03 (Sodium Nitrite)<br>D09 (Ethanolamine)<br>G08 (g−Amino−n−Butyric Acid)<br>E06 (Glucuronamide)<br>B05 (L−Leucine)<br>C10 (L−Citrulline)<br>A04 (Sodium Nitrate)<br>C04 (D−Asparagine)<br>F04 (Cytidine)<br>E08 (D−Glucosamine)<br>B11 (L−Threonine)<br>D02 (N−Phthaloyl−L−Glutamic Acid)<br>D01 (N−Acetyl−L−Glutamic Acid)<br>B06 (L−Lysine)<br>G06 (Parabanic Acid)<br>B08 (L−Phenylalanine)<br>E05 (Formamide)<br>H04 (Ala−Gly)<br>C01 (L−Tyrosine)<br>G03 (Uric Acid)<br>H10 (Gly−Glu)<br>C12 (L−Ornithine)<br>H11 (Gly−Met)<br>B02 (Glycine)<br>G12 (L−Norvaline)<br>G11 (d−Amino−Valeric Acid)<br>F01 (N−Acetyl−b−D−Mannosamine)<br>E11 (N−Acetyl−D−Glucosamine)<br>C07 (D−Lysine)<br>F05 (Cytosine)<br>F08 (Thymine)<br>F10 (Uracil)<br>F11 (Uridine)	H02 (Thiourea)<br>F01 (Negative Control #2)<br>H12 (Tetramethylene Sulfone)<br>G12 (L−Methionine Sulfone)<br>H04 (D,L−Lipoamide)<br>F11 (Cysteamine)	A12 (Adenosine−3',5'−Cyclic Monophosphate)<br>D01 (D−Mannose−1−Phosphate)<br>E09 (Thymidine−3'−Monophosphate)<br>E10 (Thymidine−5'−Monophosphate)<br>D09 (Uridine−3'−Monophosphate)<br>D08 (Uridine−2'−Monophosphate)<br>E02 (O−Phospho−L−Tyrosine)<br>E03 (Phosphocreatine)<br>E05 (O−Phosphoryl−Ethanolamine)<br>B11 (Guanosine−2',3'−Cyclic Monophosphate)<br>B10 (Guanosine−5'−Monophosphate)<br>A10 (Adenosine−5'−Monophosphate)<br>B09 (Guanosine−3'−Monophosphate)<br>D12 (Uridine−3',5'−Cyclic Monophosphate)<br>C12 (Cytidine−3',5'−Cyclic Monophosphate)<br>A04 (Trimetaphosphate)<br>A09 (Adenosine−3'−Monophosphate)<br>A11 (Adenosine−2',3'−Cyclic Monophosphate)<br>B12 (Guanosine−3',5'−Cyclic Monophosphate)<br>D03 (Cysteamine−S−Phosphate)<br>B01 (Thiophosphate #1)<br>C09 (Cytidine−3'−Monophosphate)<br>C10 (Cytidine−5'−Monophosphate)<br>D10 (Uridine−5'−Monophosphate)<br>C11 (Cytidine−2',3'−Cyclic Monophosphate)<br>D11 (Uridine−2',3'−Cyclic Monophosphate)<br>A06 (Triethyl Phosphate)<br>A07 (Hypophosphite)

Subfigure A – PM1 PM2 Carbon sources	Subfigure B - PM3 nitrogen sources	Subfigure C - PM4 Sulfur sources	Subfigure D - PM4 Phosphorus sources
F09 (Sorbic Acid)	G07 (D,L−a−Amino−Butyric Acid)		A01 (Negative Control #1)
B07 (L−Arabitol)	E10 (D−Mannosamine)		E06 (Phosphono Acetic Acid)
C10 (a−Methyl−D−Mannoside)	C08 (D−Serine)		E08 (Methylene Diphosphonic Acid)
C11 (b−Methyl−D−Xylopyranoside)	A11 (L−Cysteine)		E11 (Inositol Hexaphosphate)
B10 (m−Erythritol)	C11 (L−Homoserine)		
C06 (a−Methyl−D−Glucoside)	G10 (D,L−a−Amino−Caprylic Acid)		
C05 (Maltitol)	A06 (Biuret)		
E09 (g−Hydroxy−Butyric Acid)	E09 (D−Galactosamine)		
D08 (Xylitol)	E07 (D,L−Lactamide)		
D03 (Sedoheptulosan)	E12 (N−Acetyl−D−Galactosamine)		
C08 (3−O−Methyl−D−Glucose)	B12 (L−Tryptophan)		
C02 (L−Glucose)	G04 (Alloxan)		
G01 (Acetamide)	A01 (Negative Control)		
F12 (L−Tartaric Acid)	D04 (Hydroxylamine)		
G12 (L−Methionine)	D05 (Methylamine)		
G09 (Mono−Methyl Succinate)	D08 (Ethylamine)		
G11 (D−Malic Acid)	C05 (D−Aspartic Acid)		
F10 (Succinamic Acid)	D07 (N−Butylamine)		
A12 (Dulcitol)	E03 (Tyramine)		
H12 (3−Hydroxy−2−Butanone)	E04 (Acetamide)		
A09 (Inulin)	E02 (b−Phenylethylamine)		
B11 (D−Fucose)	B07 (L−Methionine)		
D06 (D−Tagatose)	C09 (D−Valine)		
D07 (Turanose)	D10 (Ethylenediamine)		
B04 (Amygdalin)	C06 (D−Glutamic Acid)		
C09 (b−Methyl−D−Glucuronic Acid)	D06 (N−Amylamine)		
H07 (Glucuronamide)	F09 (Thymidine)		
H05 (D−Psicose)	G09 (e−Amino−N−Caproic Acid)		
G05 (Glycine)			
F05 (Oxalomalic Acid)			
B04 (L−Fucose)			
B02 (D−Sorbitol)			
E03 (a−D−Glucose−1−Phosphate)			
F04 (D−Threonine)			
H11 (2,3−Butanedione)			
C03 (D−Lactitol)			

**Subfigure A – PM1 PM2 Carbon sources**	**Subfigure B - PM3 nitrogen sources**	**Subfigure C - PM4 Sulfur sources**	**Subfigure D - PM4 Phosphorus sources**
F10 (Glyoxylic Acid)<br>H04 (Tyramine)<br>F03 (myo−Inositol)<br>H02 (p−Hydroxy−Phenylacetic Acid)<br>G08 (N−Acetyl−b−D−Mannosamine)<br>H11 (b−Phenylethylamine)<br>H10 (2,3−Butanediol)<br>H06 (Butylamine [sec])<br>F03 (Melibionic Acid)<br>G02 (Tricarballylic Acid)<br>E09 (Adonitol)<br>F09 (Glycolic Acid)<br>D04 (1,2−Propanediol)<br>D08 (a−Methyl−D−Galactoside)<br>E06 (a−Hydroxy−Glutaric Acid−g−Lactone)<br>E08 (b−Methyl−D−Glucoside)<br>D11 (Sucrose)<br>B10 (Sodium Formate)<br>C11 (D−Melibiose)<br>D04 (L−Sorbose)<br>F04 (Oxalic Acid)<br>D05 (Stachyose)<br>E11 (Itaconic Acid)<br>D09 (N−Acetyl−D−Glucosaminitol)<br>F07 (D−Ribono−1,4−Lactone)<br>D02 (D−Aspartic Acid)<br>B03 (b−D−Allose)<br>F08 (Sebacic Acid)<br>G07 (L−Homoserine)<br>E06 (2−Hydroxy−Benzoic Acid)<br>A01 (Negative Control)<br>A01 (Negative Control).1<br>A02 (Chondroitin Sulfate C)<br>E07 (a−Hydroxy−Butyric Acid)<br>G11 (L−Lysine)<br>D01 (D−Raffinose)			

Subfigure A – PM1 PM2 Carbon sources	Subfigure B - PM3 nitrogen sources	Subfigure C - PM4 Sulfur sources	Subfigure D - PM4 Phosphorus sources
C04 (D−Melezitose)<br>D02 (D−Salicin)<br>A11 (Mannan)<br>D07 (a−Keto−Butyric Acid)<br>B12<br>(3−O−b−D−Galactopyranosyl−D−Arabinose)<br>A03 (a−Cyclodextrin)<br>A05 (g−Cyclodextrin)<br>D12 (Butyric Acid)<br>D11 (d−Amino−Valeric Acid)<br>E07 (4−Hydroxy−Benzoic Acid)<br>E01 (Capric Acid)<br>G02 (L−Alaninamide)<br>E10 (a−Keto−Valeric Acid)<br>G07 (Acetoacetic Acid)<br>H07 (D,L−Octopamine)<br>B07 (D,L−a−Glycerol−Phosphate)<br>F01 (Gly−Asp)<br>G06 (L−Histidine)<br>C12 (Thymidine)<br>E04 (D−Fructose−6−Phosphate)<br>E11 (2'−Deoxy−Adenosine)<br>C01 (D−Glucose−6−Phosphate)<br>B01 (D−Serine)<br>B09 (2−Deoxy−D−Ribose)<br>C12 (Palatinose)<br>A10 (Laminarin)<br>F11 (D−Cellobiose)<br>G04 (L−Threonine)<br>A12 (Pectin)<br>B03 (Glycerol)<br>H01 (Gly−Pro)<br>A04 (b−Cyclodextrin)<br>B01 (N−Acetyl−D−Galactosamine)<br>B08 (Arbutin)<br>C10 (D−Maltose)			

Subfigure A – PM1 PM2 Carbon sources	Subfigure B - PM3 nitrogen sources	Subfigure C - PM4 Sulfur sources	Subfigure D - PM4 Phosphorus sources
E10 (Maltotriose)<br>A07 (Gelatin)<br>A08 (Glycogen)<br>E12 (5−Keto−D−Gluconic Acid)<br>B05 (D−Arabinose)<br>A06 (Dextrin)<br>A10 (D−Trehalose)<br>H09 (Dihydroxy−Acetone)<br>H06 (L−Lyxose)<br>B02 (N−Acetyl−Neuraminic Acid)<br>C07 (b−Methyl−D−Galactoside)<br>C01 (b−Gentiobiose)<br>D09 (a−D−Lactose)<br>H03 (m−Hydroxy−Phenylacetic Acid)<br>F11 (D−Tartaric Acid)<br>C06 (L−Rhamnose)<br>D10 (Lactulose)			
